# Immune Checkpoint Molecules on Tumor-Infiltrating Lymphocytes and Their Association with Tertiary Lymphoid Structures in Human Breast Cancer

**DOI:** 10.3389/fimmu.2017.01412

**Published:** 2017-10-30

**Authors:** Cinzia Solinas, Soizic Garaud, Pushpamali De Silva, Anaïs Boisson, Gert Van den Eynden, Alexandre de Wind, Paolo Risso, Joel Rodrigues Vitória, François Richard, Edoardo Migliori, Grégory Noël, Hugues Duvillier, Ligia Craciun, Isabelle Veys, Ahmad Awada, Vincent Detours, Denis Larsimont, Martine Piccart-Gebhart, Karen Willard-Gallo

**Affiliations:** ^1^Molecular Immunology Unit, Institut Jules Bordet, Université Libre de Bruxelles, Brussels, Belgium; ^2^Department of Pathology, GZA Ziekenhuizen, Wilrijk, Belgium; ^3^Department of Pathology, Institut Jules Bordet, Brussels, Belgium; ^4^Health Sciences Department – DISSAL, University of Genova, Genova, Italy; ^5^IRIBHM, Bioinformatics Laboratory, Université Libre de Bruxelles, Bruxelles, Belgium; ^6^Breast Cancer Translational Laboratory, Institut Jules Bordet, Université Libre de Bruxelles, Brussels, Belgium; ^7^Flow Cytometry Facility, Institut Jules Bordet, Brussels, Belgium; ^8^Department of Surgery, Institut Jules Bordet, Brussels, Belgium; ^9^Department of Medicine, Institut Jules Bordet, Brussels, Belgium

**Keywords:** PD-1, PD-L1/PD-L2, CTLA-4, LAG3, TIM3, tumor-infiltrating lymphocytes, tertiary lymphoid structures, breast cancer

## Abstract

There is an exponentially growing interest in targeting immune checkpoint molecules in breast cancer (BC), particularly in the triple-negative subtype where unmet treatment needs remain. This study was designed to analyze the expression, localization, and prognostic role of PD-1, PD-L1, PD-L2, CTLA-4, LAG3, and TIM3 in primary BC. Gene expression analysis using the METABRIC microarray dataset found that all six immune checkpoint molecules are highly expressed in basal-like and HER2-enriched compared to the other BC molecular subtypes. Flow cytometric analysis of fresh tissue homogenates from untreated primary tumors show that PD-1 is principally expressed on CD4^+^ or CD8^+^ T cells and CTLA-4 is expressed on CD4^+^ T cells. The global proportion of PD-L1^+^, PD-L2^+^, LAG3^+^, and TIM3^+^ tumor-infiltrating lymphocytes (TIL) was low and detectable in only a small number of tumors. Immunohistochemically staining fixed tissues from the same tumors was employed to score TIL and tertiary lymphoid structures (TLS). PD-L1^+^, PD-L2^+^, LAG3^+^, and TIM3^+^ cells were detected in some TLS in a pattern that resembles secondary lymphoid organs. This observation suggests that TLS are important sites of immune activation and regulation, particularly in tumors with extensive baseline immune infiltration. Significantly improved overall survival was correlated with PD-1 expression in the HER2-enriched and PD-L1 or CTLA-4 expression in basal-like BC. PD-1 and CTLA-4 proteins were most frequently detected on TIL, which supports the correlations observed between their gene expression and improved long-term outcome in basal-like and HER2-enriched BC. PD-L1 expression by tumor or immune cells is uncommon in BC. Overall, the data presented here distinguish PD-1 as a marker of T cell activity in both the T and B cell areas of BC associated TLS. We found that immune checkpoint molecule expression parallels the extent of TIL and TLS, although there is a noteworthy amount of heterogeneity between tumors even within the same molecular subtype. These data indicate that assessing the levels of immune checkpoint molecule expression in an individual patient has important implications for the success of therapeutically targeting them in BC.

## Introduction

Targeting the immune checkpoint molecules CTLA-4, PD-1, and PD-L1 is revolutionizing clinical management for a variety of solid and hematological malignancies by generating durable anti-tumor immune responses (1, 2). The success of these drugs is nevertheless currently limited to a subset of patients whose tumors likely produce neoantigens and/or whose anti-tumor responses are constrained by immune inhibitory pathways. Immune stimulatory/co-stimulatory pathways are tightly regulated by feedback inhibitory pathways that limit normal immune responses to prevent excessive activity. Thus, molecules controlling these pathways are being actively investigated to identify new and potentially more refined targets for cancer immunotherapy.

The tumor immune microenvironment varies considerably between solid tumor types as well as within tumors of the same organ or tissue developing in different individuals. There is a general consensus that chronic inflammation promotes tumor progression with tumors at diagnosis ranging from a baseline inflamed phenotype to an immunological desert (3). In breast cancer (BC), a variety of studies have investigated the clinical value of tumor-infiltrating leukocytes [CD45^+^ cells that are >90% lymphocytes] (4) and immune gene signature expression (5–8) with significant findings obtained for HER2-positive (HER2^+^) and triple negative (TN) BC. Our recent studies show that tumor-infiltrating lymphocytes (TIL) density in a cohort of BC patients (all subtypes) whose tumors were analyzed within hours of surgery forms a continuum (9). Using a threshold defined by normal breast tissues, we identified 25% of tumors as TIL-negative (TIL^neg^). TIL-positive tumors (TIL^pos^; 75%), equally divided into TIL-intermediate (TIL^int^) and TIL-high (TIL^hi^), were defined by a second threshold set based on non-adjacent non-tumor tissue from the same BC patients. We further found a positive correlation between the extent of TIL, the presence of organized tertiary lymphoid structures (TLS), and PD-1^+^ T cell TIL and/or PD-L1^+^ immune cells (7, 9, 10). Our data reveal significant heterogeneity and complexity in the adaptive anti-tumor immune response, which suggests that BC stratification based on the balance of TIL subpopulations and/or TLS might be clinically relevant and identify patients most likely to respond to immune checkpoint inhibitors.

We previously reported that TLS are principally located in the stroma of up to 60% of BC (7, 9). TLS were initially identified in lung (11) and colorectal (12) cancer but have now been associated with positive clinical outcomes in an expanding number of solid tumors (13). TLS architecture is characterized by a T cell zone adjacent to a B cell follicle similar to secondary lymphoid organs (SLOs) and mucosal-associated lymphoid tissues (e.g., tonsils). An active germinal center (GC) is often detected in the B cell follicle, providing a site for B cell differentiation and maturation into antibody producing plasmacytes or memory B cells. Specialized CD4^+^ T cells named T follicular helper cells (Tfh) play a key role in GC formation and B cell differentiation/maturation. Our previous work identified PD-1^hi^CD200^hi^ Tfh cells as an important component of BC TIL and linked their presence with tumor-associated TLS and good clinical outcomes, particularly in HER2^+^ and TNBC (7). Tfh cells have been identified in other solid tumors (14), including pancreatic adenocarcinoma where TLS with GC were characterized by PD-1^+^ TIL in 32/104 patients (15). Our latest study identified a subpopulation of CXCR5^−^PD-1^hi^ICOS^int^CD4^+^ Tfh TIL (named TfhX13 cells) that produce the chemoattractant CXCL13 and thereby recruit CXCR5^+^ T and B cells to TLS (10). Interestingly, we also detected a FOXP3^hi^CXCR5^−^PD-1^int^ICOS^hi^CD4^+^ TIL subpopulation, which is primarily regulatory T cells (Tregs), whose presence is linked with TfhX13 TIL accumulation. Together, our data suggest that PD-1/PD-L1 expression in BC may reflect ongoing, active immune responses rather than (or in addition to) immunosuppression *via* this pathway.

PD-1 is an important immune checkpoint molecule, which together with its principal ligand PD-L1, is actively targeted in the clinic. Despite promising initial outcomes in melanoma, lung, and kidney cancer (16, 17), the clinical benefit of immune checkpoint inhibitors in BC has been restricted to a subset of patients and effective predictive biomarkers remain undefined (18). Clinically, PD-1 expression in BC has been associated with good (7, 19) or bad (20) outcomes. Similarly, PD-L1 expression was associated with a good (19, 21–23) or bad prognosis (24–26). No clinical significance has been attributed to expression of the alternate PD-1 ligand PD-L2 (27). The first immune checkpoint molecule targeted in the clinic, CTLA-4, was analyzed in BC with the presence of CTLA-4^+^, PD-1^+^, Helios^+^, FOXP3^+^, GITR^+^, and/or CD103^+^ Tregs suggestive of an immunosuppressive microenvironment (28, 29).

CTLA-4 expression on tumor cells was associated with a bad prognosis (30) while good outcomes were observed when CTLA-4^+^ TIL were present (19, 31, 32).

The remarkable responses of immunotherapeutic agents in some patients is driving early-phase clinical studies designed to broaden their efficacy and benefit by combining agents that target non-redundant inhibitory pathways. LAG3, which inhibits the activity of MHC-II^+^ antigen presenting cells (APCs), was evaluated in a retrospective cohort of TNBC with 18% positivity detected; however, its expression was not correlated with outcome despite a trend toward a better prognosis for LAG3-positivity (33). Another target of clinical interest is TIM3, a molecule that curbs effector T cell activation after binding to galectin-9 on immune cells as well as stromal and tumor cells (34, 35). Interestingly, TIM3^+^ Tfh cells from BC patient blood were shown to have reduced functionality (36). Numerous ongoing clinical trials are combining agents targeting the PD-1/PD-L1 pathway with those targeting CTLA-4, LAG3, or TIM3.

The genetic and immune heterogeneity of BC together with the restricted number of patients that currently derive benefit from immunotherapy exposes an urgent need to improve patient selection for immunotherapy. The purpose of this study was to evaluate expression of PD-1, PD-L1, PD-L2, CTLA-4, LAG3 and TIM3 in the primary BC microenvironment. A combination of flow cytometry (FACS) and immunohistochemistry (IHC) allowed us to quantify their expression in fresh tissue homogenates coupled with a determination of their spatial location in the tumor.

## Materials and Methods

### Human Samples

A well-annotated cohort including 95 untreated primary invasive breast carcinomas collected from female patients diagnosed and treated at the Institut Jules Bordet in Brussels and the GZA Ziekenhuizen in Antwerp between March 2015 and December 2016 was analyzed. These breast tumors include 34% luminal A (*N* = 32), 33% luminal B (*N* = 31), 20% HER2^+^ (*N* = 19), and 13% TNBC (*N* = 13) based on routine analysis of immunohistochemically stained sections (Table S1 in Supplementary Material). All specimens and clinical data were analyzed using procedures approved by the Institut Jules Bordet’s Medical Ethics Committee (CE 2403) and the GZA Ziekenhuizen Medical Ethics Committee (CE 130909ACADEM). All patients signed their informed consent.

### Gene Expression Microarray Data

Public microarray data were derived from the METABRIC dataset (37) using normalized expression data available from the European Genome Archive (Accession number EGAS00000000083; discovery set plus validation set, total *N* = 1986, 6 uncategorized tumors were excluded). The expression of each gene was evaluated for PAM50 defined BC molecular subtype. All analyses were done using the open source statistical language R (38).

### TIL Isolation and Analysis

TIL in fresh breast tissues from untreated primary BC were analyzed by FACS as previously published (39). Multi-color FACS was performed using antibodies to well-defined immune cell markers, including CD3, CD4, CD8, CD19 and CD45 and a panel of immune checkpoint molecules: PD-1, PD-L1, PD-L2, CTLA-4, LAG3, and TIM3 (details of the antibodies are provided in Table S2A in Supplementary Material). Intracellular CTLA-4 (iCTLA-4) was labeled by fixing and permeabilizing membrane-labeled cells with the BD Cytofix/Cytoperm™ Fixation/Permeabilization Solution Kit (BD 554714) following the manufacturer’s instructions. Sample data were acquired on a GALLIOS 10/3 cytometer and analyzed using Kaluza^®^ 1.3 Flow Analysis Software (Beckman Coulter, Brea, CA, USA). *N* = *X* in the data plots indicate the number of analyzable tumors for a given TIL marker (tumors with insufficient FACS data acquisition were excluded). The amount of material for experimental analysis was limited by the routine requirements of the pathology laboratory, which explains why it was not always possible to analyze all markers for each sample.

### Immunohistochemistry

Immunohistochemistry staining of formalin-fixed paraffin embedded (FFPE) tumor tissues (4 µm sections) was performed on a Ventana Benchmark XT IHC/ISH automated staining instrument (Ventana Medical Systems) with the antibodies detailed in Table S2B in Supplementary Material. Tonsils were used as controls, described in Ref. (9). Single IHC stains were performed for PD-L2, LAG3, and TIM3 using the UltraView Universal DAB Detection Kit (Ventana Medical Systems, Inc., Tucson, AZ, USA). Dual IHC staining procedures have been previously described (9). Ki67, scored as the fraction (percentage) of Ki67-positive cells, was derived from the original pathology report; stromal TIL and intratumoral TIL were defined using published guidelines (40) and our published methodology (41). Global TIL were calculated as the sum of %stromal TIL plus %intratumoral divided by 2. CD3, CD20, CD4, and CD8 were calculated as the fraction (percentage) of positive cells in the tumor including both stroma and intratumoral areas. TLS were defined as areas of dense B cell follicles (CD20^+^ cells) surrounded by T cells (CD3^+^ cells) as previously published (41). The number of TLS was normalized to the tumor area and multiplied by 100. Reading was done independently by two experienced immunopathologists (Gert Van den Eynden and Alexandre de Wind) who were blinded to the clinical data.

### Immunofluorescent Confocal Microscopy

Immunofluorescence (IF) staining was performed manually on FFPE tumor tissues [4 µm sections; detailed in Ref. (42)]. After washing, slides were mounted with ProLong Gold anti-fade mounting medium with DAPI (Thermo Scientific) and visualized on a Zeiss LSM 710 confocal microscope equipped with a ×20/0.8 Plan-Apochromat dry objective (Carl Zeiss, Oberkochen, Germany). The primary and secondary antibodies used for IF confocal microscopy are detailed in Table S2C in Supplementary Material.

### Statistical Analysis

Gene expression analysis was conducted using normalized logarithmic base 2 (log2) values. Dunn’s multiple comparison tests were performed to compare the median log expression of each marker for each PAM50 BC molecular subtype. *P*-values ≤0.05 were considered statistically significant. All analyses were done using the open source statistical language R (38). FACS data are presented for median, minimum, maximum, and means ± SD values. Analyses on continuous variables were conducted using the Spearman correlation. Fisher exact test computed the contingency tables based on categorical data. All reported *P*-values were two-tailed and a test comparison was considered statistically significant if the associated *P*-value was ≤0.05. The Benjamini–Hochberg procedure was used for the *P*-value correction (43). Statistical analyses were performed using the SAS BASE version 9.4. The intraclass correlation coefficient (ICC) was used to measure the inter-observer variance for scores from the two immunopathologists (Table S3 in Supplementary Material). ICC was calculated using the mixed model to assess the reliability of pathologists scoring averaged together based on the Shrout and Fleiss definition (44). This was performed using the open source statistical language R (38). Hazard ratios (HRs) for overall survival (OS) and disease-specific survival (DSS) were calculated using the Cox’s proportional hazards model (45). Proportional hazard assumptions were tested and *P*-values were computed and summarized in the forest plots. HRs were calculated for log2 expression data for the markers of interest (per 1 unit of fold change). These analyses were performed using the open source statistical language R (38).

## Results

### The Immune Infiltrate in a Prospective Cohort of Patients with Primary BC

The extent and composition of the immune infiltrate in primary BC was evaluated on dual IHC-stained sections using our previously published methodology (41). This approach was shown to produce accurate and reproducible scoring by experienced immunopathologists of TIL and TLS in the tumor microenvironment (41). The present study scored stromal, intratumoral, and global TIL (as a percentage of the defined area), TLS as well as CD3^+^, CD4^+^, CD8^+^, and CD20^+^ TIL as a percentage of the defined stromal, tumor, and global areas (Table S1 in Supplementary Material). The ICC ranged between 0.74 and 0.94 for the variables of interest (Table S3 in Supplementary Material) with mean scores used for data analyses. In this BC cohort, the extent of TIL was associated with histological grade, the presence of *in situ* carcinoma, estrogen receptor (ER), and progesterone receptor (PR) negativity, a TLS presence and PD-L2-positivity after adjustment for multiple comparisons (Table S4 in Supplementary Material). A TLS presence was consistently and significantly associated with baseline TIL (including CD4^+^, CD8^+^ and CD20^+^ subpopulations) and ER-negativity (ER^neg^) (adjusted for multiple comparisons).

### Comparative Analysis of PD-1, PD-L1, and PD-L2 Expression and Correlation with Clinicopathological Parameters in Primary BC

**Figure 1:**
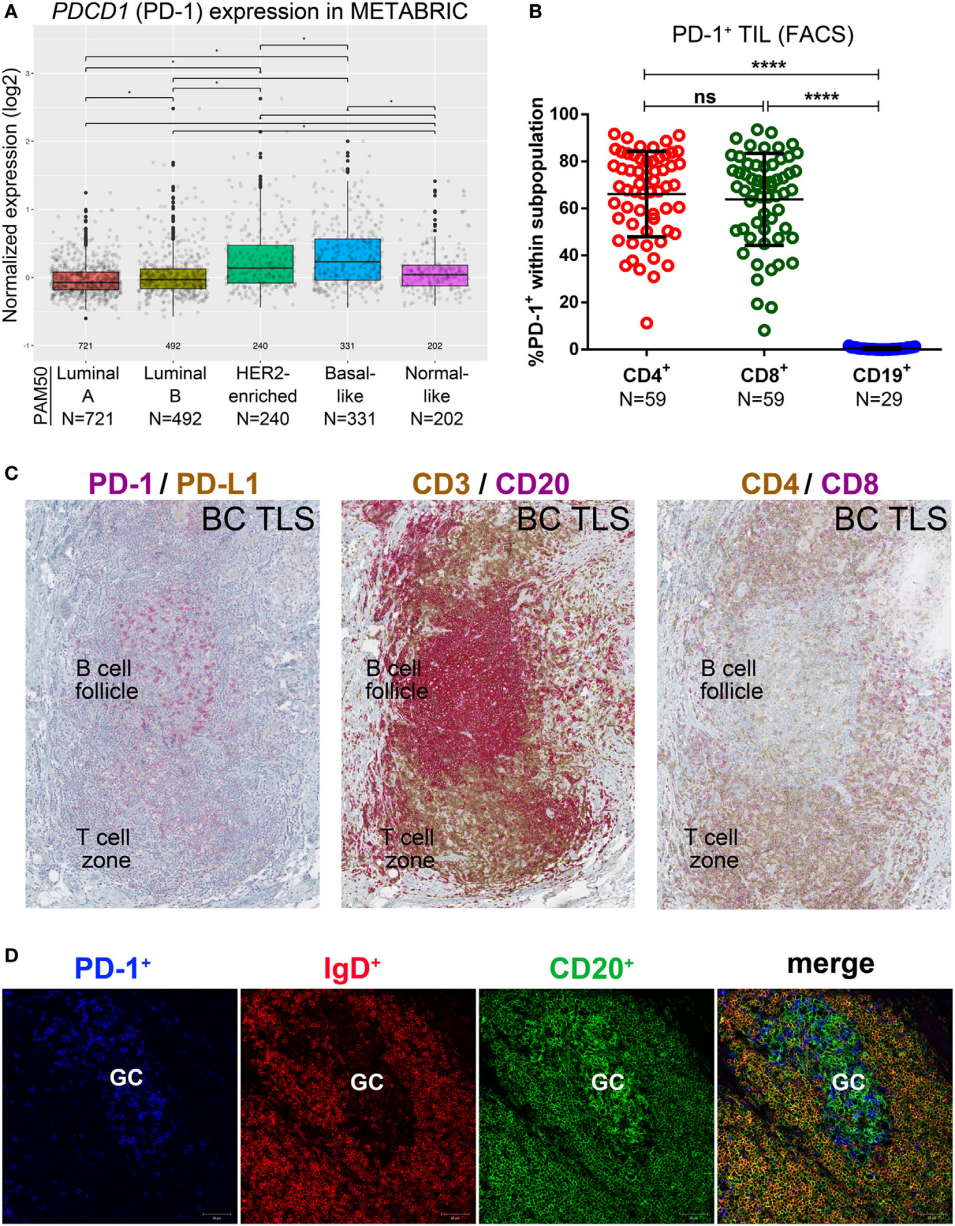
PD-1 expression in primary breast cancer (BC). **(A)** Expression of the *PDCD1* gene in BC by PAM50 molecular subtypes (microarray data from the METABRIC dataset) (37). Box plots show median ± interquartile range related to log of expression of each gene by different subtypes (Luminal A, Luminal B, HER2-enriched, Basal-like and Normal-like). **(B)** PD-1 expression by the major subsets (CD4^+^, CD8^+^, and CD19^+^) of tumor-infiltrating lymphocytes (TIL) isolated from fresh primary breast tumors analyzed by FACS. Bars show mean ± SD. The number of samples included (*N*) is based on the tumor homogenate having a threshold minimum number of TIL for analysis. **(C)** Expression of PD-1 and PD-L1 by immunohistochemistry in BC-associated tertiary lymphoid structures (TLS) stained with dual CD3/CD20, CD4/CD8, and PD-L1/PD-1 labeling. In brown: CD3, CD4 and PD-L1; in red: CD20, CD8 and PD-1. **(D)** Expression of PD-1, IgD, and CD20 by immunofluorescence in the germinal center (GC) of a BC associated TLS. In blue: PD-1, in red: IgD, in green CD20.

**Figure 2:**
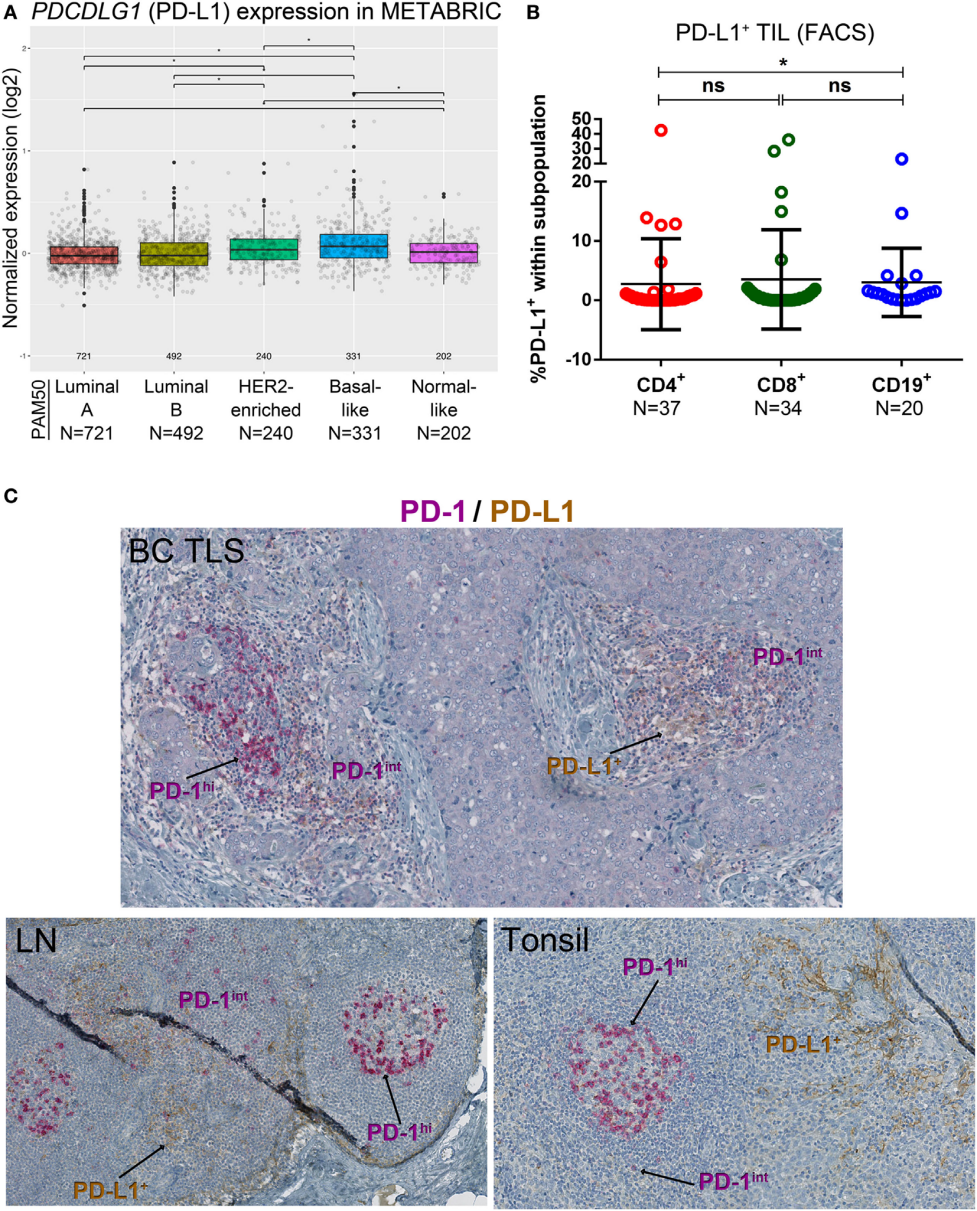
PD-L1 expression in primary breast cancer (BC). **(A)** Expression of the *PDCDLG1* gene in BC by PAM50 molecular subtypes (microarray data from the METABRIC dataset) (37). Box plots show median ± interquartile range related to log of expression of each gene by different subtypes (Luminal A, Luminal B, HER2-enriched, Basal-like, and Normal-like). **(B)** PD-L1 expression by the major subsets (CD4^+^, CD8^+^, and CD19^+^) of tumor-infiltrating lymphocytes (TIL) isolated from fresh primary breast tumors analyzed by FACS. Bars show mean ± SD. The number of samples included (*N*) is based on the tumor homogenate having a threshold minimum number of TIL for analysis. **(C)** Expression of PD-L1 and PD-1 by immunohistochemistry in BC-associated tertiary lymphoid structures (TLS), in a normal human lymph node (LN) and in a human tonsil. In brown: PD-L1; in red: PD-1.

**Figure 3:**
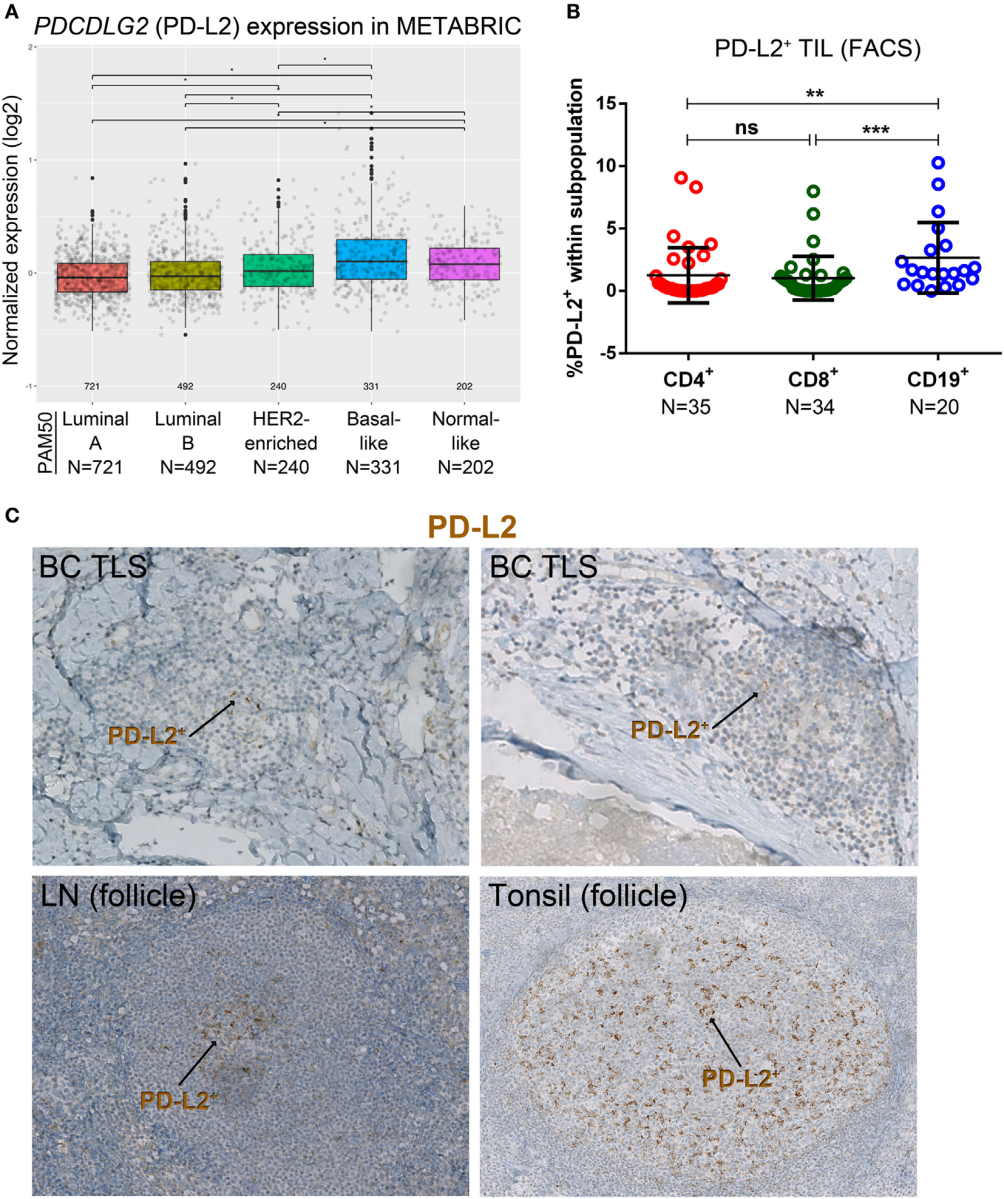
PD-L2 expression in primary breast cancer (BC). **(A)** Expression of the *PDCDLG2* gene in BC by PAM50 molecular subtypes (microarray data from the METABRIC dataset) (37). Box plots show median ± interquartile range related to log of expression of each gene by different subypes (Luminal A, Luminal B, HER2-enriched, Basal-like, and Normal-like). **(B)** PD-L2 expression by the major subsets (CD4^+^, CD8^+^, and CD19^+^) of tumor-infiltrating lymphocytes (TIL) isolated from fresh primary breast tumors analyzed by FACS. Bars show mean ± SD. The number of samples included (*N*) is based on the tumor homogenate having a threshold minimum number of TIL for analysis. **(C)** Expression of PD-L2 by immunohistochemistry in BC-associated tertiary lymphoid structures (TLS), in a normal human lymph node (LN), and in a human tonsil. In brown: PD-L2.

A variety of technical approaches was used to examine the expression and location of PD-1 and its ligands, PD-L1 and PD-L2 in primary BC. First, the publically available METABRIC dataset (37) was used to interrogate *PDCD1* (PD-1), *PDCDLG1* (PD-L1), and *PDCDLG2* (PD-L2) gene expression in 1,896 human BC stratified by molecular subtype using PAM50 (38). PD-1 is more highly expressed in basal-like compared with the other BC subtypes; in HER2-enriched compared to luminal A/B and normal-like; in normal-like compared to luminal A/B; and in luminal B compared to luminal A (Figure 1A). PD-L1 is also expressed at higher levels in basal-like compared to the other BC molecular subtypes; in HER2-enriched compared to luminal A/B and normal-like; and in normal-like compared to luminal A (Figure 2A). PD-L2 gene is more expressed in basal-like compared to HER2-enriched and luminal A/B; in HER2-enriched compared to luminal A/B; and in normal-like compared to luminal A/B and HER2-enriched (Figure 3A). Next, TIL in fresh breast tissues from untreated primary BC were analyzed by FACS within a few hours of surgery using procedures previously described (39). Analysis of PD-1 expression on CD4^+^ and CD8^+^ TIL reveals that >1% of TIL are PD-1^+^ in all of the samples analyzed and as expected, CD19^+^ B cells are negative (Figure 1B; Table S5 in Supplementary Material). In some tumors, three subpopulations expressing high, intermediate, and low levels of PD-1 (PD-1^hi^, PD-1^int^, and PD-1^lo^) were detected on CD4^+^ and CD8^+^ TIL, with these categories most distinct on CD4^+^ TIL (Figure S1A in Supplementary Material). A small proportion of BC TIL (principally from extensively infiltrated HER2^+^ and TNBC) expresses both ligands (PD-L1 and PD-L2) on their cell surfaces (Figures 2B and 3B; Figures S2A and S3A and Tables S5, S7B, and S8B in Supplementary Material).

The percentage of PD-1^+^CD4^+^ TIL determined by FACS was positively correlated with IHC scores for %Ki67, %stromal TIL, %global TIL, %intratumoral TIL, %CD4, %CD8, and %CD20 (Table S6A in Supplementary Material). No significant correlations were observed between %PD-1^+^CD8^+^ TIL and IHC scores; however, PD-1^hi^CD8^+^ TIL (FACS) are associated with tumor size, *in situ* carcinoma, menopausal status, PD-1^hi^CD4^+^ TIL, and PD-L2 status (Table S6B in Supplementary Material). The presence of PD-1^hi^CD4^+^ TIL (FACS) was associated with histotype (ductal *versus* others) and high CD20^+^ B cell TIL (IHC). Although trends were found, these findings were not statistically significant after correction for multiple comparisons, likely due to our cohort size. Overall, we show that PD-1 is exclusively expressed on T cells and PD-1^hi^CD8^+^ TIL are linked with PD-1^hi^CD4^+^ TIL and PD-L2 expression on TIL in BC. The association of PD-1^hi^CD4^+^ TIL with high CD20^+^ B cell TIL likely reflects their Tfh phenotype (7, 10).

The percentage of PD-L1^+^ cells in CD4^+^ and CD8^+^ TIL was positively correlated with tumor size, IHC scores for %stromal TIL, %global TIL, %intratumoral TIL, and %CD8^+^, even if only a low proportion of PD-L1^+^ TIL was detected (Figure 2B; Table S5 in Supplementary Material). Significant correlations were also observed between PD-L1^+^CD8^+^ TIL and TLS, %CD4^+^ and %CD20^+^, with a trend toward a significant association detected for CD4^+^ TIL in the same categories (Table S7A in Supplementary Material). As a continuous variable, PD-L1^+^CD19^+^ B TIL were positively correlated with age but no other significant findings were observed. As a categorical variable, PD-L1^+^ TIL (defined as >1% PD-L1^+^ cells in any TIL subset) were associated with tumor size, in the presence of an iCTLA-4^hi^CD4^+^ TIL subpopulation and PD-L2^+^ TIL (defined as >1% PD-L2^+^ cells in any TIL subset) (Table S7B in Supplementary Material). After correction for multiple comparisons, these data did not achieve statistical significance (although trends were found) likely because of the cohort size.

The percentage of PD-L2^+^ TIL, as a continuous variable in both CD4^+^ and CD8^+^ TIL, was positively correlated with tumor size and IHC scores for %Ki67, %stromal TIL, %global TIL, %intratumoral TIL, TLS, %CD4^+^, and %CD8^+^ (Table S8A in Supplementary Material) despite a very low proportion of positivity. Significant correlations or trends toward a positive correlation were observed for PD-L2^+^ TIL in both CD4^+^ and CD8^+^ TIL analyzed by FACS and %CD20^+^ TIL scored by IHC, respectively. No significant correlations were found for PD-L2^+^ TIL in CD19^+^ TIL as a continuous variable, even though a trend toward significance was observed for %CD4 (IHC scores). The prevalence of PD-L2^+^ expression on any TIL was associated with high %Ki67, ER^neg^ and PR^neg^ BC, the extent of TIL infiltration, a TLS presence, PD-1^hi^ CD8^+^ TIL, LAG3^+^ TIL (>1% LAG3^+^ cells in any TIL subset), and PD-L1^+^ TIL (Table S8B in Supplementary Material). Again despite trends, after multiple comparison corrections statistical significance was not achieved, likely due to the cohort size. Overall, the data presented here confirm and extend our recent findings linking PD-1^+^ TIL and PD-L1 expression [IHC (9)], by acquiring PD-1, PD-L1 and PD-L2 FACS expression analysis of fresh tissues and correlating these data with the extent of TIL and a TLS presence and other clinicopathological parameters in a new, independent prospective series of untreated primary BC.

### Tissue Sites of PD-1^+^, PD-L1^+^, and PD-L2^+^ TIL in BC

PD-1^+^ TIL are often localized in the B cell follicle of a TLS, as previously shown (9, 10, 15). Closer examination of these PD-1^+^ TIL in our BC cohort reveals that they can also be relatively dense in the T cell zone of a TLS, which is primarily composed of CD4^+^ and CD8^+^ TIL (Figure 1C). To gain further insight, we comparatively analyzed dual PD-1 and PD-L1 IHC-stained tissue sections (Figure 1C; whole tumor sections are shown in Figure S1B in Supplementary Material) with IF-stained tissue sections examined by confocal microscopy (Figure 1D). These data show that PD-1^hi^ TIL are particularly dense in the GC (Figures 1C,D) of a TLS B cell follicle, supporting their identity as PD-1^hi^CD4^+^ Tfh TIL (7, 10, 15). PD-1^int^ TIL can be found in the TLS T cell zone. TLS containing PD-1^hi/int^ TIL are most often found in TIL^hi^ BC, which are frequently HER2^+^ or TNBC (Table S4 in Supplementary Material) (7, 9, 10).

Our previous work detected PD-L1 on tumor, stromal, and immune cells in IHC-stained tissue sections of untreated primary BC (9). The dual PD-1 and PD-L1 IHC stain allowed us to compare their relative expression in BC TLS with SLOs [lymph node (LN) and tonsil tissues; Figure 2C]. Interestingly, across TLS^+^ tumors with extensive immune infiltrates, we observed a pattern: PD-1^hi^ TIL in the GC (majority CD4^+^ T cells) (7, 10), PD-1^int^ TIL in the T cell zone, and occasionally PD-L1 expression was detected within a TLS. These findings parallel SLOs where PD-1^hi^ cells are concentrated in the GC (Figure 2C) and PD-L1^+^ cells and PD-1^int^ cells are occasionally present in the intrafollicular spaces. PD-L1^+^ cells are also detected in the marginal sinus of LNs and the reticulated epithelium of tonsillar crypts. Morphologically, most of the PD-L1^+^ cells (BC and SLOs) appear to be large cells from the myeloid lineage. The alternate PD-1 ligand, PD-L2, was densest on APC resident in tonsil GCs (Figure 3C) while its expression in LNs was significantly lower and essentially limited to the center of a GC. PD-L2^+^ BC tissue sections contained only a few TLS with positively stained areas at the center of a GC. The expression of this ligand was weak or absent in most of the tumors we examined, even those with extensive baseline TIL infiltration and a high TLS score.

The prognostic significance of *PDCD1* (PD-1), *PDCDLG1* (PD-L1), and *PDCDLG2* (PD-L2) gene expression in primary BC was evaluated using the METABRIC dataset for OS and DSS (Figures S7A–C, S8A–C, S9A–C, and S10A–C in Supplementary Material). The multivariate analyses show that PD-1 expression was associated with significantly improved OS and DSS in the HER2-enriched subtype; PD-L1 expression with improved OS and DSS in the basal-like subtype and a worse OS in the normal-like subtype; PD-L2 expression with better DSS in the basal-like subtype. Taken together, these data show that while PD-1 is readily expressed on BC TIL, the expression of its ligands is infrequent with PD-L1 more frequently detected than PD-L2 and predominantly in the basal-like subtype. Despite a paucity of these ligands, a low proportion of PD-L1 or PD-L2 on TIL (FACS analysis) appears to signal a baseline anti-tumor immune response characterized by extensive TIL infiltration and TLS (detectable by IHC). This suggests that their presence is linked with active anti-tumor immunity.

### CTLA-4^+^ Expression on TIL and Its Correlation with Clinicopathological Parameters in Primary BC

**Figure 4:**
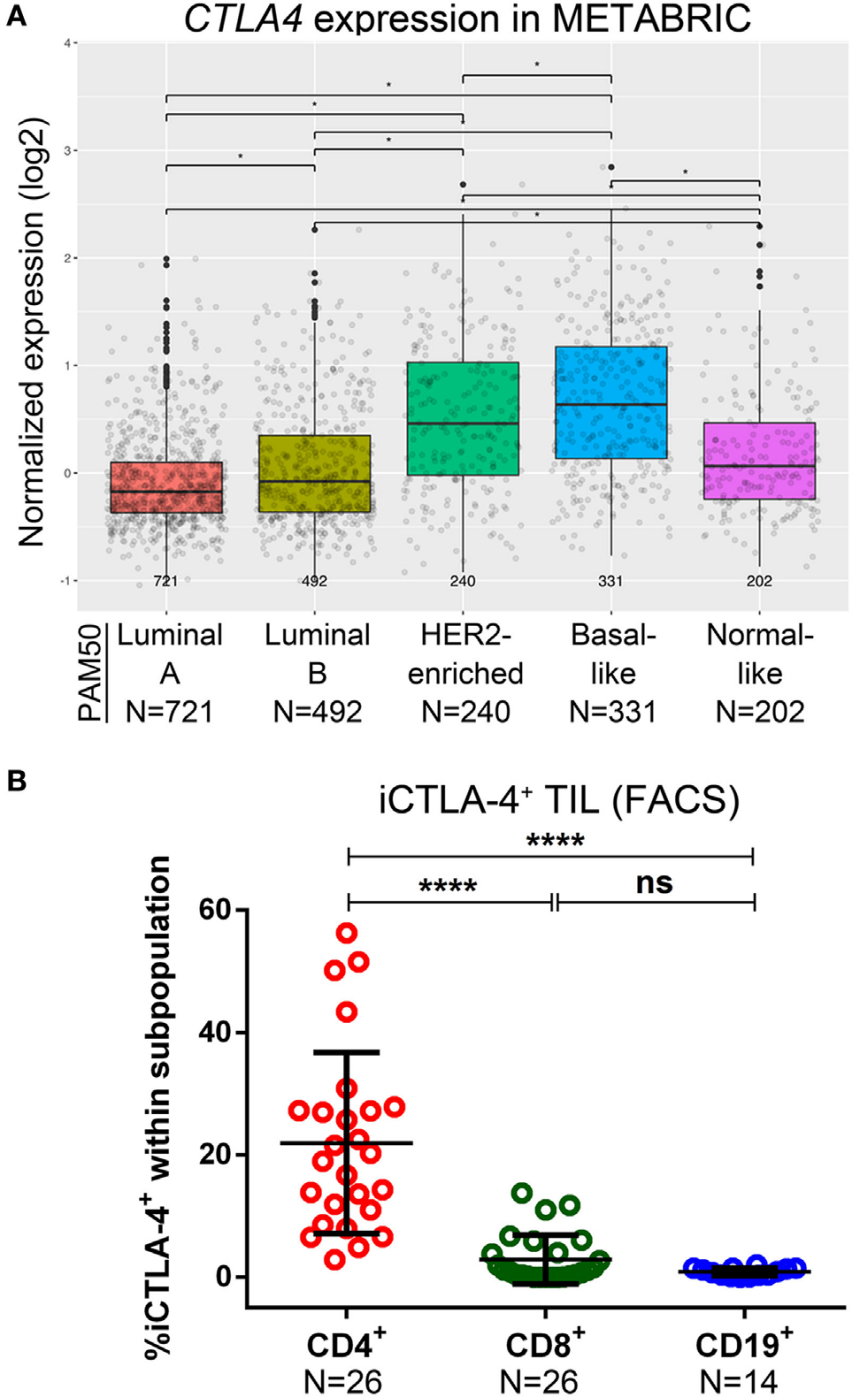

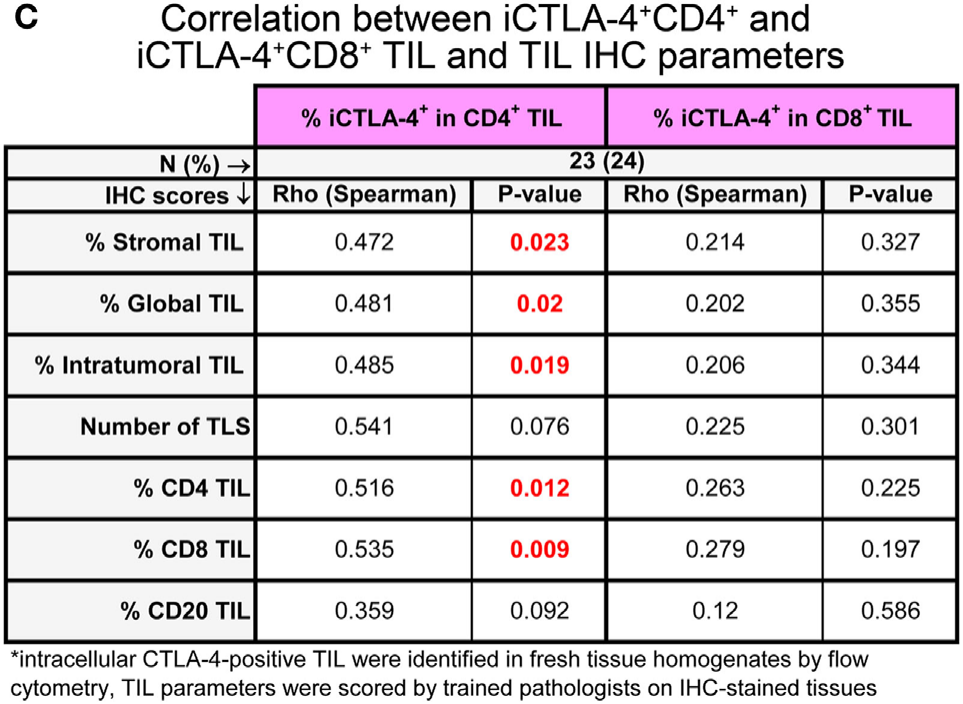
Expression of CTLA-4 in primary breast cancer. **(A)** Expression of the *CTLA4* gene by PAM50 molecular subtypes (microarray data from the METABRIC dataset) (37). Box plots show median ± interquartile range related to log of expression of *CTLA4* gene in different subtypes (Luminal A, Luminal B, HER2-enriched, Basal-like, and Normal-like). **(B)** Intracellular CTLA-4 (iCTLA-4) expression by the major subsets (CD4^+^, CD8^+^, and CD19^+^) of tumor-infiltrating lymphocytes (TIL) isolated from fresh primary breast tumors analyzed by FACS. Bars show mean ± SD. The number of samples included (*N*) is based on the tumor homogenate having a threshold minimum number of TIL for analysis. **(C)** Correlations between the percentage of iCTLA-4 in CD4^+^ and CD8^+^ TIL (evaluated by FACS) and immune infiltration continuous parameters evaluated by immunohistochemistry (IHC).

Analysis of METABRIC gene expression data (37) shows that in primary BC, *CTLA4* is more frequently expressed in the basal-like and HER2-enriched subtypes compared with luminal A, luminal B and normal-like, and in luminal B compared to luminal A; in normal-like compared to luminal A/B (Figure 4A). CD4^+^ TIL were the major iCTLA-4^+^ subpopulation although occasional iCTLA-4^+^CD8^+^ TIL were detected (Figure 4B; Figure S4A and Table S5 in Supplementary Material) (46). The %iCTLA-4^+^CD4^+^ TIL (FACS) was positively correlated with IHC scores (FFPE tissue from the same tumors) for %stromal TIL, %global TIL, %intratumoral TIL, %CD4^+^, and %CD8^+^ (Figure 4C; Table S9A in Supplementary Material). A trend toward significance was associated with a TLS presence and the %CD20. We categorized the iCTLA-4 in CD4^+^ TIL variable using a 75^th^ percentile cut-off (iCTLA-4^hi^) to investigate potential associations with clinicopathological parameters. The prevalence of iCTLA-4^hi^ within CD4^+^ TIL was associated with ER^neg^ and PR^neg^ BC, the IHC clinical subtypes, a TLS presence and PD-L1^+^ TIL (Table S9B in Supplementary Material), although these findings were not statically significant after *P*-value corrections for multiple testing. A trend toward significance was detected between iCTLA-4^hi^CD4^+^ TIL and the extent of TIL infiltration. The presence of *in situ* carcinoma was associated with iCTLA-4^low/int^CD4^+^ TIL (iCTLA-4^+^ TIL below the 75^th^ percentile). No significant correlations were observed between %iCTLA-4^+^CD8^+^ TIL as a continuous variable and the immune infiltration parameters evaluated by IHC (Figure 4C; Table S9A in Supplementary Material). While these data show that iCTLA-4 is principally expressed on CD4^+^ TIL in HER2^+^ and TNBC with TIL^int^ to TIL^hi^ infiltration levels, analysis of a larger cohort is needed. Examination of the prognostic significance of *CTLA4* gene expression in the METABRIC dataset found an association with significantly improved OS and DSS in the basal-like BC molecular subtype using multivariate analyses (Figures S7D, S8D, S9D, and S10D in Supplementary Material).

### LAG3 Expression on Immune Cells Infiltrating Primary BC

**Figure 5:**
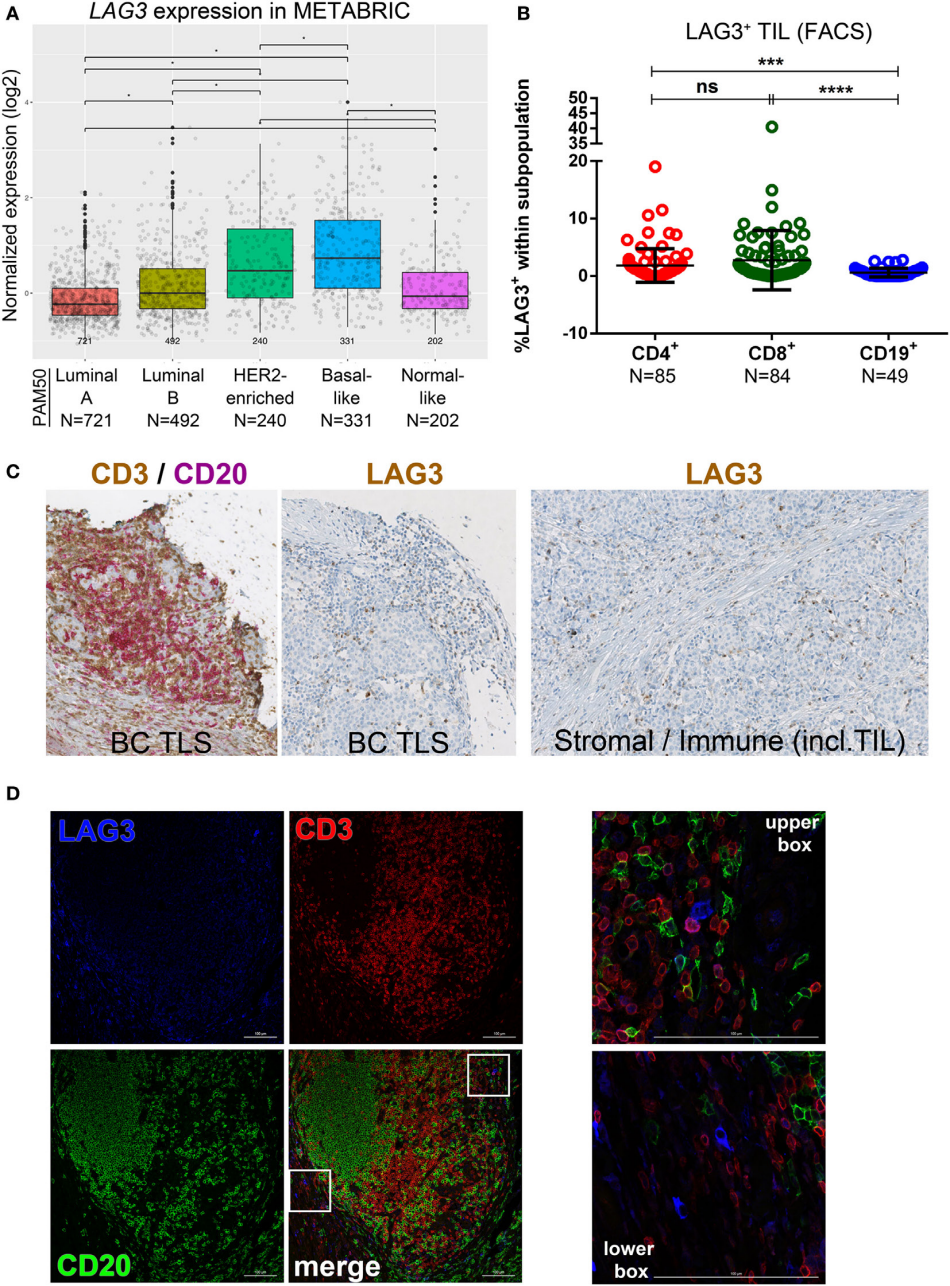
Expression of LAG3 in primary breast cancer (BC). **(A)** Expression of *LAG3* gene by PAM50 molecular subtypes (microarray data from the METABRIC dataset) (37). Box plots show median ± interquartile range related to log of expression of *LAG3* gene in different subtypes (Luminal A, Luminal B, HER2-enriched, Basal-like, and Normal-like). **(B)** LAG3 expression by the major subsets (CD4^+^, CD8^+^, and CD19^+^) of tumor-infiltrating lymphocytes (TIL) isolated from fresh primary breast tumors analyzed by FACS. Bars show mean ± SD. The number of samples included (*N*) is based on the tumor homogenate having a threshold minimum number of TIL for analysis. **(C)** LAG3 expression in BC-associated tertiary lymphoid structures (TLS) by immunohistochemistry (IHC). A representative example of a TLS stained with the dual CD3/CD20 and with the LAG3 IHC stain. In brown: CD3 and LAG3; in red: CD20. LAG3 was also found expressed by immune cells including TIL and plasmocytes localized in the stroma or in intratumoral areas and by stromal cells (i.e., fibroblasts, etc.). **(D)** Immunofluorescence staining of a BC associated TLS with LAG3 (in blue), CD3 (in red), and CD20 (in green). In the upper box: LAG3^+^ CD3^+^ TIL; in the lower box: non-lymphoid LAG3^+^ cells.

Gene expression analysis using the METABRIC dataset (37) reveals that *LAG3* is more frequently detected in the basal-like compared with the other subtypes and in the HER2-enriched BC subtype compared with luminal A/B and normal-like; in luminal B compared to luminal A; and in normal-like compared to luminal A (Figure 5A). A low proportion of LAG3^+^ cells were detected in CD4^+^ and CD8^+^ TIL with CD19^+^ TIL-negative for this marker (Figure 5B; Figure S5A and Table S5 in Supplementary Material). The percentage of LAG3^+^ in CD4^+^ or CD8^+^ TIL, as continuous variables, was positively correlated with %Ki67, %stromal TIL, %global TIL, TLS, %CD4^+^, %CD8^+^, and %CD20^+^ scored on IHC-stained sections (Table S10A in Supplementary Material). The prevalence of LAG3^+^ BC (defined as >1% CD4^+^ or CD8^+^ LAG3^+^ TIL by FACS) was associated with ER^neg^, the clinical subtypes TNBC and HER2^+^, and a presence of TIM3^+^ or PD-L2^+^ TIL (Table S10B in Supplementary Material); however, after correcting for multiple testing, these results were not statistically significant, again likely due to sample size. LAG3 expression was not significantly associated with OS or DSS in any BC subtype after multivariate analyses (Figures S7E, S8E, S9E, and S10E in Supplementary Material).

Close examination of LAG3 IHC-stained tissue sections reveals predominant surface membrane expression; however, trans-Golgi vesicles containing LAG3 are occasionally detectable in the cytoplasm (47). Lymphocytes are the principal LAG3^+^ population but this receptor is also expressed on other immune cells [i.e., plasmacytoid dendritic cells (DCs)] (Figure 5C) (48). LAG3^+^ T cell TIL were dispersed in the stroma or in the TLS T cell zone, similar to human tonsils where its expression predominates in the T cell zones and is only occasionally detected in GC (Figure S5B in Supplementary Material). In our cohort, breast tumor cells did not express LAG3. IF analysis identified LAG3^+^ T cell TIL principally at the periphery of TLS T cell zones in extensively infiltrated tumors (Figure 5D), which is similar to tonsil tissues (Figure S5C in Supplementary Material). LAG3 expression was also detected on CD3^−^CD20^−^ immune cells, which are potentially natural killer or plasmacytoid DCs (Figures 5D). These data suggest that LAG3 expression, highest in TNBC and HER2^+^ primary BC, is restricted to a small proportion of stromal or TLS T cells where LAG3^+^ stromal and immune cells are also found. A consistent characteristic of LAG3^+^ BC is extensive immune infiltration and the expression of other immune checkpoint molecules, including PD-L2 and TIM3.

### Cells in the Primary BC Microenvironment That Express TIM3

**Figure 6:**
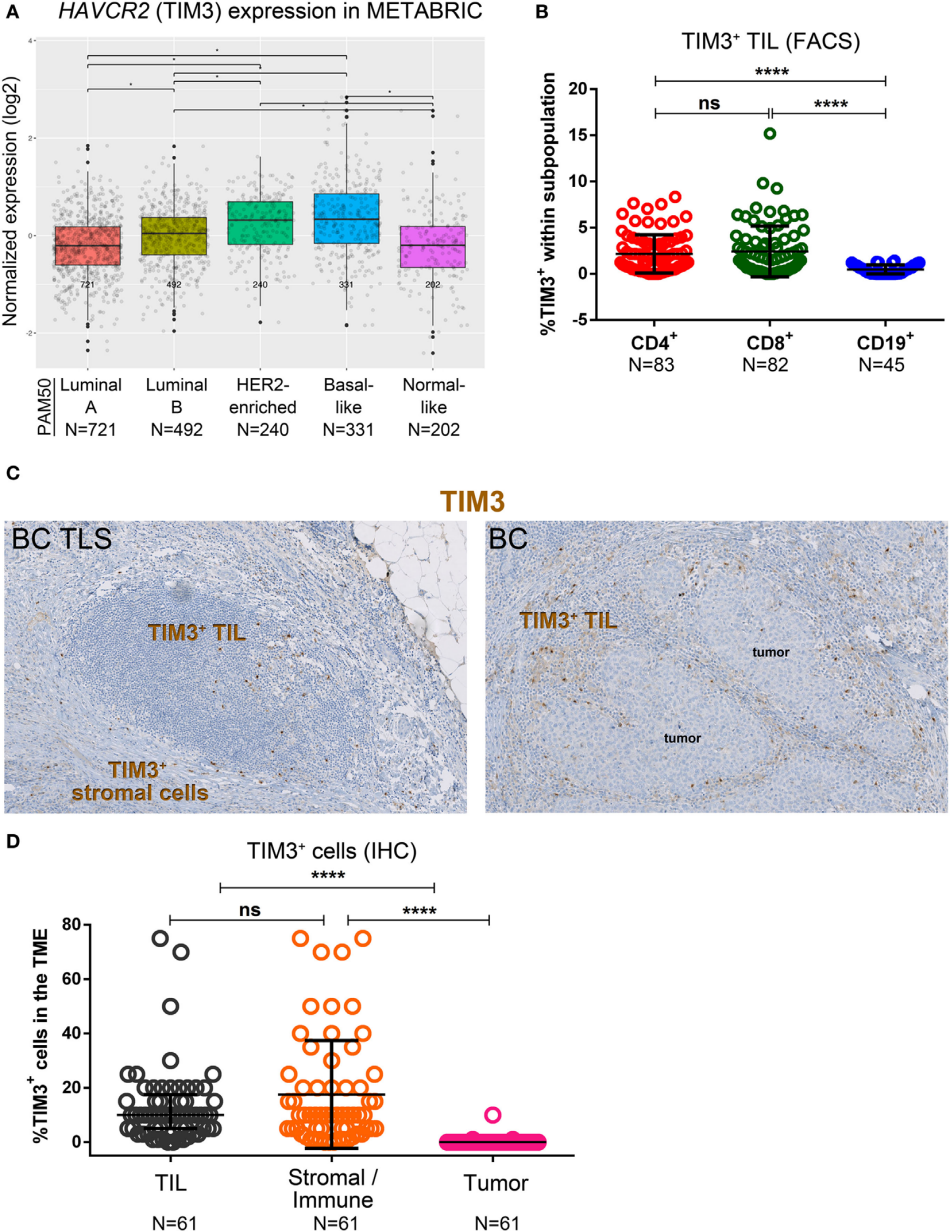
Expression of TIM3 in primary breast cancer (BC). **(A)** Expression of *HAVCR2 (TIM3)* gene by PAM50 molecular subtypes (microarray data from the METABRIC dataset) (37). Box plots show median ± interquartile range related to log of expression of *HAVCR2 (TIM3)* gene in different subtypes (Luminal A, Luminal B, HER2-enriched, Basal-like and Normal-like). **(B)** TIM3 expression by the major subsets (CD4^+^, CD8^+^, and CD19^+^) of tumor-infiltrating lymphocytes (TIL) isolated from fresh primary breast tumors analyzed by FACS. Bars show mean ± SD. The number of samples included in the analysis (*N*) is based on a minimum number of cells in each TIL subpopulation. **(C)** TIM3 expression in BC-associated tertiary lymphoid structures (TLS) and in BC TIL and stromal cells by immunohistochemistry (IHC). **(D)** Expression of TIM3 on stromal cells, tumor cells, and TIL by IHC scored by an immunopathologist. Bars show mean ± SD and **** refers to a *P* value <0.0001.

The *HAVCR2* gene, which encodes TIM3, is more highly expressed in basal-like and HER2-enriched compared with luminal A/B and normal-like and in luminal B compared to luminal A and normal-like BC subtypes in the METABRIC dataset (Figure 6A) (37). FACS analysis of fresh BC tissues reveals that TIM3 is expressed only on a small fraction of CD4^+^ and CD8^+^ TIL (Figure 6B; Table S5 in Supplementary Material) with CD19^+^ B cell TIL TIM3-negative (Figure S6A in Supplementary Material). TIM3^+^CD8^+^ TIL were significantly correlated with a TLS presence (Table S11A in Supplementary Material), with a similar trend [close to statistical significance (*P* = 0.052)] observed for TIM3^+^CD4^+^ TIL. The categorical TIM3^+^ variable (defined as the presence of >1% TIM3^+^ TIL by FACS) was associated with the presence of LAG3^+^ TIL and had borderline statistical significance with PD-L1^+^ TIL (*P* = 0.0562, uncorrected) (Table S11B in Supplementary Material). These findings were not confirmed by correcting for multiple testing. Multivariate analyses did not find a significant impact for *HAVCR2* (TIM3) gene expression in the primary BC subtypes for OS and DSS (Figures S7F, S8F, S9F, and S10F in Supplementary Material).

Immunohistochemistry staining for TIM3 revealed that not only it is expressed on TIL but also on stromal and other immune cells (i.e., histiocytes) (Figure 6C), while tumor cells are TIM3-negative (Figure 6D). Consecutive tumor sections IHC-stained for CD68 (macrophage marker) and TIM3 suggest that the “other immune cells” are TIM3^+^ macrophages (Figure S6C in Supplementary Material). In TLS, TIM3 expression is usually detected on T cell zone TIL, similar to tonsils (Figure S6B in Supplementary Material), with some expression also on immune cells localized in the TLS (Figure 6C). Our data show that TIM3 expression in primary BC is higher in basal-like and HER2-enriched BC. Perhaps more importantly, TIM3 expression is heterogeneous and while it is mainly detected on a small proportion of TLS-resident T cells, it is also associated with LAG3^+^ and PD-L1^+^ TIL. Thus, the variety of different cell types expressing TIM3 complicates our understanding of the role it plays in regulating the BC microenvironment.

## Discussion

The work presented in this study is the first, to our knowledge, to characterize a panel (PD-1, PD-L1, PD-L2, CTLA-4, LAG3, and TIM3) of targetable inhibitory immune checkpoint molecules in primary BC. This analysis was undertaken to (1) correlate their expression in the BC molecular subtypes; (2) associate specific BC cellular subpopulations with clinicopathological data; (3) situate their location in the tumor microenvironment; and (4) correlate their presence with BC prognosis. Overall, immune checkpoint molecule expression was significantly higher in basal-like (~80% TNBC) and HER2-enriched (~80% HER2^+^) compared to the other BC molecular subtypes. PD-1 and CTLA-4 were uniquely expressed on T cell TIL, while LAG3, TIM3, PD-L1, and PD-L2 were detected at low levels on TIL in tumors with extensive infiltration and TLS. PD-L1, LAG3, and TIM3 were also expressed on stromal and other immune cells in the tumor microenvironment. PD-1, PD-L1, and CTLA-4 gene expression was significantly associated with improved clinical outcome in basal-like and HER2-enriched BC. These data are consistent with previous studies using immune gene signatures (5–7, 49) and/or evaluating the overall presence of TIL (50–52). Our observation that this panel of immune checkpoint molecules is predominantly expressed on TIL suggests that their presence and abundance parallels the TIL continuum we recently established for primary BC (9). These data also reveal a contrast between BC and solid tumor types where checkpoint molecules are frequently expressed on tumor cells, such as melanoma, renal, and lung cancer (16, 17). This study validates the clinical value of a preexisting active immune response at the tumor site, particularly in TN and HER2^+^ BC (4).

Our analysis of TIL in freshly prepared tissues together with their corresponding FFPE blocks produced some new and interesting findings. We found no differences in the proportion of PD-1^+^ TIL within the CD4^+^ or CD8^+^ compartments in the 56 primary BC analyzed. We further show that PD-1 expression is heterogeneous on both CD4^+^ and CD8^+^ T cell TIL with PD-1^hi^, PD-1^int^, and PD-1^neg^ subpopulations distinguishable by FACS, particularly in tumors with extensive TIL and TLS. Heterogeneity in the degree of PD-1 expression between CD4^+^ T cell subpopulations could reflect CD4^+^ TIL with different functional profiles (i.e., PD-1^hi^CD4^+^ = Tfh; PD-1^int^CD4^+^ = Treg, PD-1^neg^CD4^+^ = naïve T cells, etc.). We recently identified PD-1^hi^ICOS^int^CD4^+^ TIL as a subpopulation containing effector helper (Th) cells and specialized CXCL13-producing Tfh cells, called TfhX13, which are thought to be important mediators of TLS formation and immune cell recruitment (10). We also showed that PD-1^int^ICOS^hi^CD4^+^ TIL contain a preponderance of FOXP3^hi^ Tregs. Our observation that PD-1^hi^ICOS^int^ and PD-1^int^ICOS^hi^ CD4^+^ TIL are linearly correlated in 90% of BC, suggests that effector Th plus TfhX13 TIL frequently expand in parallel with activated Treg TIL (10), thereby generating anti-tumor immunity and memory that is regulated by immune feedback mechanisms. Following this rationale, an imbalance favoring regulatory cells would promote an immunosuppressive state that essentially disables active immune responses. In a recent study of claudin-low BC (a TNBC subset), investigators found that a substantial proportion of TIL in these aggressive tumors are Tregs. They further demonstrated that significant Treg depletion together with checkpoint inhibition was necessary for effective anti-tumor immunity (53). The role of Tregs in BC remains controversial with both positive and negative effects on outcome reported, potentially because their ratio to effectors is an important influence on immune activity and clinical outcome. This idea is supported by a study showing that higher ratios of FOXP3^+^CD4^+^ to CD8^+^ T cells in biopsies from BC patients with ductal carcinoma *in situ* predict their relapse (54). These investigators also observed that the same high ratio in adjacent normal tissue was predictive of poor outcomes.

PD-1^hi^CD8^+^ and PD-1^int^CD8^+^ TIL were detected in all of the TIL^int^ and TIL^hi^ primary BC we analyzed although their proportions varied. While a functional role for these CD8^+^ T cell subpopulations is currently unknown, CD8^+^PD-1^int^ TIL are thought to be more responsive to PD-1 or PD-L1 blocking agents (55). A separate study found that high PD-1 and PD-L1 expression in pretreatment biopsies was significantly correlated with higher TIL and higher rates of pathological complete response after neoadjuvant treatment (44). Further investigation is needed to determine whether the balance between PD-1 (PD-1^hi^, PD-1^int^ or PD-1^lo^) CD4^+^ and CD8^+^ TIL subpopulations is linked with clinical responses to checkpoint inhibitors targeting the PD-1/PD-L1 pathway.

The PD-1^hi^ or PD-1^int^ TIL subpopulations we identified by FACS (10) (Figure S1 in Supplementary Material) were also detected on dual PD-1/PD-L1 IHC-stained sections (Figure 2C). Interestingly, the PD-1^int^ TIL are often observed adjacent to areas containing PD-L1^+^ cells similar to SLO tissues. We further found that PD-1^hi^ TIL are principally located in the GC of a TLS while the PD-1^int^ TIL are outside the GC but primarily within a TLS or TIL aggregate. Taken altogether, these data suggest that PD-1 expression reflects an activated T cell presence in the tumor microenvironment; however, there are inconsistent findings between tumor types. In lung tumors with extensive CD8^+^ TIL, two groups were identified: (1) low immune checkpoint molecule expression, mature DCs located in TLS, and a good prognosis or (2) high checkpoint expression associated with a lack of mature DCs and an increased risk of relapse (56). In pancreatic cancer, B cells were localized in the tumor bed and TLS, with the latter alone predicting longer survival (15). Interestingly, a B cell TLS presence was associated with a GC gene signature, correlated with CD8^+^ TIL and a positive prognosis in these patients. Future studies using multi-plex IHC should help to precisely establish critical location-based interactions and balances between checkpoint molecules on effector and regulatory TIL that drive immune activation or suppression and help predict clinical responses.

An initial FACS study of PD-L1 expression on TIL and epithelial cells examined four tumors with 2/4 defined as positive (24). The present study analyzed both PD-1 ligands, PD-L1 and PD-L2, on fresh tissues from a substantially larger cohort of primary BC patients. Both studies found a low prevalence of PD-L1 positive TIL. Our previous study using IHC to examine full tumor sections found that PD-L1 expression is detected predominantly in TNBC and positively correlated with the extent of TIL and a peri-tumoral TLS presence (9). In addition to its expression on TIL and tumor cells, PD-L1 is also found on other immune cells, CD68^+^ tumor-associated macrophages and myeloid cells, such as follicular dendritic cells resident in TLS. Our results from the METABRIC gene expression analysis reveal that PD-L1 expression is associated with a better OS in basal-like BC, which is likely linked with the good prognostic value of higher TIL in this subtype. PD-L1 expression in primary BC has been widely investigated at both the RNA (57, 58) and protein (9, 24, 57, 59–63) levels. The study by Ali et al. (57) is noteworthy because it examined PD-L1 expression in 3,916 BC, finding 6% PD-L1^+^ immune cells (235/3,916) and 1.7% PD-L1^+^ tumor cells (66/3,916) by IHC. These percentages are lower than other studies presumably because IHC was performed on tissue arrays derived from archival samples, where there is an increased risk of PD-L1 degradation. Overall, the relationship between PD-L1 expression and BC prognosis remains an open question, with some studies correlating it with improved survival (57–59, 62, 64) and others not (24, 62, 63). These inconsistencies should be resolved once a standardized IHC assay (65) becomes more widely employed.

These observations reveal that PD-L1 expression is infrequent in BC with the highest prevalence observed in TNBC. Consequently, TNBC is the target of most current BC immunotherapy trials with anti-PD-1 and anti-PD-L1 agents demonstrating the best clinical benefits (although limited to a subset of patients) when given in the advanced and neoadjuvant settings (18). Tumor responses were highest in TNBC for first-line treatment of metastatic disease or when CD8^+^ TIL were present or if PD-L1 positivity was detected. At ESMO 2017, Loi et al. presented data that stromal TIL, assessed on hematoxylin and eosin-stained metastatic tissues (including metastatic LNs), were higher in treatment responders, particularly if anti-PD-1 (pembrolizumab) was given in the first line (66). Their analyses included two metastatic TNBC cohorts: one pre-treated for metastatic disease and not selected based on PD-L1 expression and the other untreated for metastatic disease and PD-L1 positive (KEYNOTE-086; monotherapy with anti-PD-1). Interestingly, stromal TIL were independent predictors of response to anti-PD-1 while PD-L1 positivity did not add any predictive value.

Examination of the other PD-1 ligand revealed that while PD-L2^+^ TIL were seldom detected in BC, their presence was associated with PD-1^hi^CD8^+^, PD-L1^+^, and LAG3^+^ TIL. Expression of PD-L2 in tumors is rare and modest, principally concentrated in the B cell follicle similar to SLOs, where it has been previously shown to be typically expressed on activated APC (DCs and macrophages) (67). In contrast to tonsils, where PD-L2 expression is intense and scattered throughout the follicle, the few positive breast tumors we observed had weaker staining with only a few positive cells, like LNs and spleen. Previous studies found PD-L2 negativity in 49% of BC (*N* = 192) (27) or 20% of TNBC and HER2^+^ BC (*N* = 25) (68). The first study based positivity on nuclear and cytoplasmic staining while the second study was based on nuclear staining alone potentially explaining their differences in frequency. We scored positivity as membrane staining based on our analysis of SLOs, with normal breast and other healthy tissues consistently negative. Based on our data, PD-L2 does not appear to be a promising target for BC immunotherapy; however, additional tumors need to be analyzed to clarify discrepancies between studies.

CTLA-4 expression, principally on CD4^+^ T cells, was heterogeneous among our BC subtypes with CTLA-4^hi^CD4^+^ TIL most frequently identified in TNBC (71%) and HER2^+^ (60%) BC. This potentially is a direct reflection of Treg abundance, an idea supported by our recent data showing PD-1^int^iCTLA-4^+^FOXP3^+^CD4^+^ TIL are associated with extensive infiltration and TLS in BC (10). These data are supported by several studies, including an analysis of >100 treatment naïve BC patients where Treg abundance in the tumor microenvironment was characterized by high levels of surface CCR8, CTLA-4, and PD-1 (69). Another study similarly found that FOXP3^+^Helios^+^CTLA-4^hi^PD-1^hi^CD4^+^ TIL characterize Tregs infiltrating BC (28). Overall, current findings indicate that dual checkpoint inhibition targeting CTLA-4 and PD-1 potentially has important synergistic effects due to their distinct regulatory activities (70).

LAG3 expression was associated with PD-L2^+^ and TIM3^+^ TIL in our cohort, which is in line with current thinking that LAG3 positivity reflects an ongoing, complex immune response regulated by several regulatory pathways (71). A recent study of two independent cohorts (*N* = 259 and *N* = 104) found 15% of early-stage TNBC expressed both PD-1 and LAG3 (33). In this study, PD-1^+^ and LAG3^+^ TIL were positively correlated with the extent of infiltration, particularly CD8^+^ TIL. The METABRIC dataset did not show any significant impact on survival in primary BC molecular subtypes; however, these survival data need confirmation from independent cohorts. A phase I trial where a LAG3 agonist was administered with paclitaxel detected increased activation of APC, NK, and CD8^+^ effector/memory cells and a 50% objective response rates in patients with advanced luminal BC (72). Currently, there are a number of trials evaluating anti-LAG3 agents in association with PD-1 or PD-L1 blocking agents in many advanced solid tumors including BC (71).

The data presented here show LAG3^+^ TIL are associated with TIM3^+^ TIL, and TIM3^+^CD4^+^ and TIM3^+^CD8^+^ TIL are marginally positively correlated with TLS. An earlier study of TIM3 in BC found higher expression on CD8^+^ TIL from primary tumors compared to their blood counterparts (73). Zhu et al. recently found TIM3 expression on Tfh cells (CXCR5^+^ICOS^+^ CD4^+^ T cells) from BC patient blood at the same frequency as healthy control blood; however, they identified higher numbers of TIM3^+^ Tfh cells in patient blood (36). These blood TIM3^+^ Tfh cells were less potent at stimulating naïve B cells *in vitro*, suggesting a possible dysfunctional profile in this subpopulation. They further showed that TIM3^+^ Tfh TIL were higher frequencies than in the patient’s blood. Our laboratory identified Tfh TIL as a hallmark of TLS with concentrations of PD-1^hi^ Tfh cells characterizing TLS with a GC (7, 10). Zhu et al. (36) were able to rescue Tfh cell functions (IL-21 and CXCL13 expression) by blocking TIM3, suggesting this as a potential mechanism for regulating Tfh-mediated activities in TLS. In another study, CD8^+^ TIL cultured with IL-15 expressed higher levels of TIM3^+^, while inhibiting TIM3 and IL-15 induced CD8^+^ TIL proliferation and IFNγ production (73). We show here that in addition to lymphocytes, TIM3 is expressed on cells from the myeloid lineage, such as macrophages and stromal cells, which could contribute to more widespread inhibition in the tumor microenvironment [reviewed in Ref. (74)]. Targeting TIM3 offers a promising new direction for immune checkpoint inhibition *via* synergistic interactions between inhibitory signals from the microenvironment and TIL.

Our data show that the extent of TIL and their organization in TLS are linked with expression of immune checkpoint molecules in BC, particularly in the TNBC and HER2^+^ subtypes, which are more frequently extensively infiltrated. Many TLS^+^ tumors had expression of one or more immune checkpoint molecules suggesting multiple anti-tumor immune responses at different stages of activity occur simultaneously in the tumor microenvironment. These responses are likely regulated by factors and cells in their immediate vicinity. Our observations in BC TLS paralleled SLOs (tonsils and LNs) suggesting that checkpoint pathways are influenced and functioning in response to normal immune control mechanisms. The positive correlation and association we found between immune checkpoint molecule expression and baseline TIL and TLS indicates that evaluation of these parameters in BC patients might identify tumors more likely to respond to immune modulating therapies.

## Ethics Statement

This study was carried out in accordance with the recommendations of the Institut Jules Bordet and GZA Ziekenhuizen medical ethics committees with written informed consent from all subjects. All subjects gave written informed consent in accordance with the Declaration of Helsinki. The protocol was approved by the Institut Jules Bordet and GZA Ziekenhuizen medical ethics committees.

## Author Contributions

CS and KW-G conceived the research and designed the experiments; CS performed the majority of experiments with critical support from SG, PS, AB, EM, GN, and HD; CS collected the clinical data; GE, AW, and DL performed the pathological evaluations; PR, JV, FR, and VD performed the statistical and public data analyses; LC, IV, and DL provided the patient samples; AA and MP-G. proposed important concepts; KW-G. supervised the research; CS and KW-G. analyzed and interpreted the data and wrote the manuscript; all authors provided advice on the final manuscript.

## Conflict of Interest Statement

The present research was conducted in the absence of any commercial or financial relationships. Authors and their institutions did not receive any payment or services from a third party for any aspect of the submitted work. No financial relationships exist with entities that could have influenced or given the appearance of potentially influencing what we wrote in the submitted work.
