# Supplementary material for: Immune Checkpoint Molecules on Tumor-Infiltrating Lymphocytes and Their Association with Tertiary Lymphoid Structures in Human Breast Cancer
Source: Front Immunol. 2017 Oct 30;8:1412. doi: 10.3389/fimmu.2017.01412 (PMC5670348; doi:10.3389/fimmu.2017.01412)
Supplement: Supplementary file 12 [file Presentation_1.PDF]

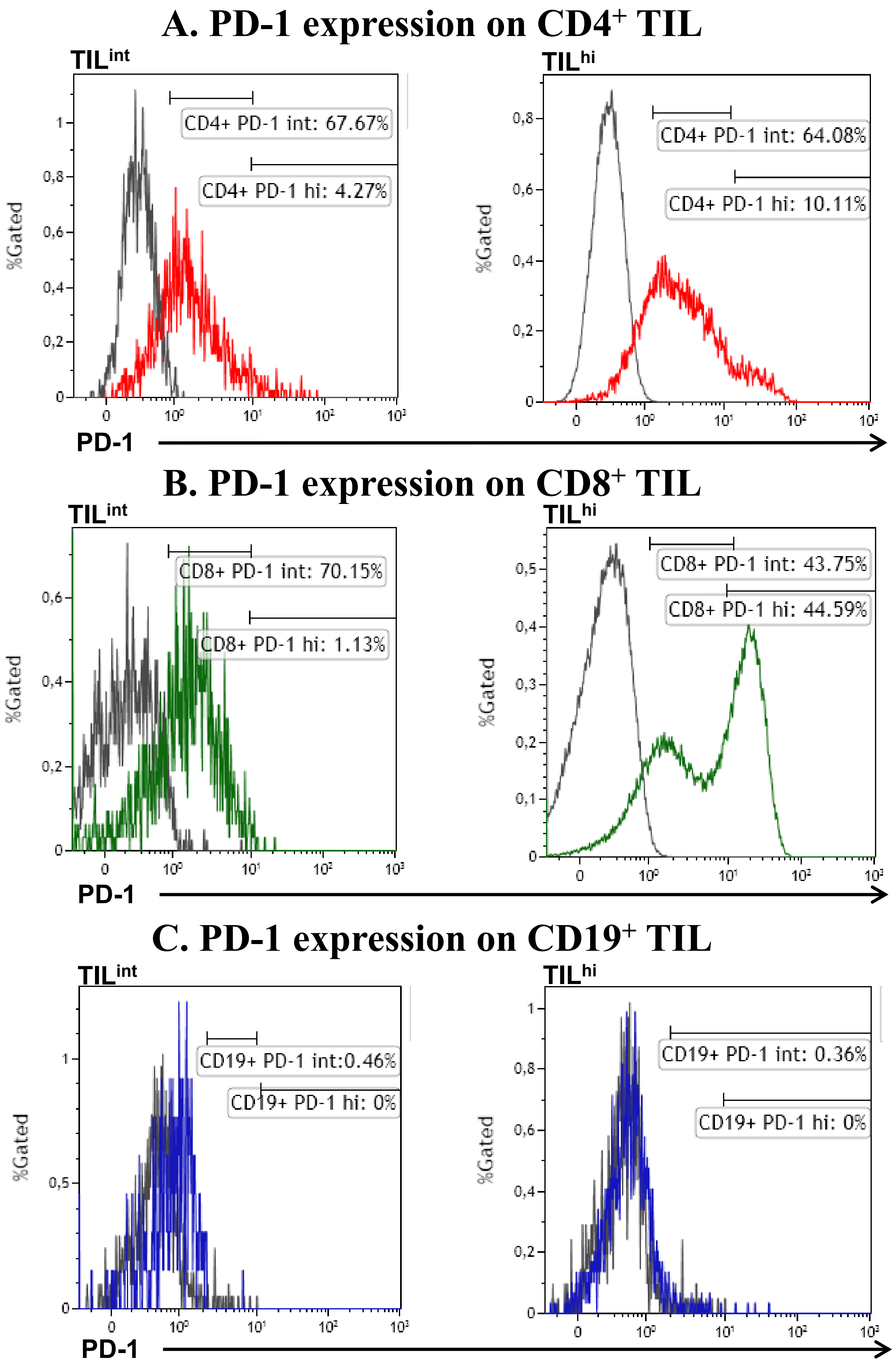

Figure S1A: PD-1 expression on lymphocyte subpopulations in breast tumors with intermediate (TIL^{int}) and high (TIL^{hi}) tumor infiltrating lymphocytes (TIL). (A) CD4^{+} T cells (red); (B) CD8^{+} T cells (green); (C) CD19^{+} B cells (blue); and isotype controls (gray in A, B and C).

Breast cancer tissue stained for PD-1/PD-L1, CD3/CD20 and CD4/CD8

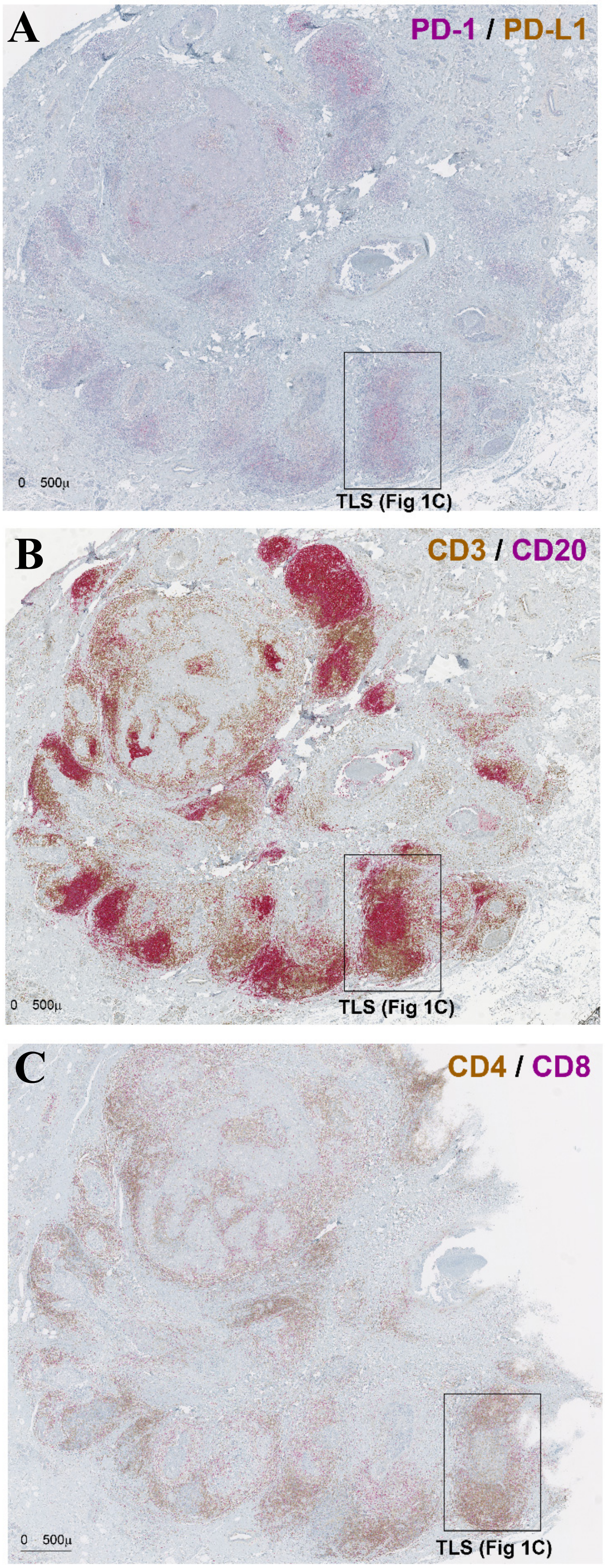

<u>Figure S1B</u>: Staining of a TIL^{hi} TNBC showing the tertiary lymphoid structure (TLS) depicted in Figure 1C. (A) dual PD-1 (red) and PD-L1 (brown) IHC; (B) dual CD3 (brown) and CD20 (red) IHC; and (C) dual CD4 (brown) and CD8 (red) IHC.

PD-L1 expression on CD4$^+$, CD8$^+$ and CD19$^+$ TIL

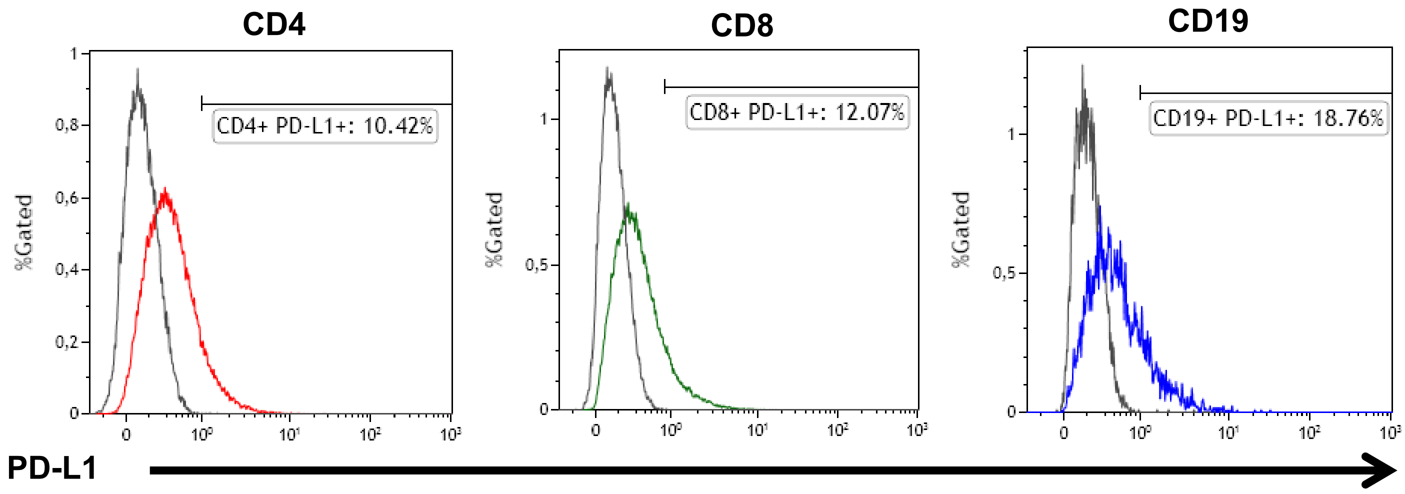

<u>Figure S2</u>: PD-L1 expression on tumor infiltrating lymphocytes (TIL) in breast tumors (a TIL^{hi} TNBC is shown) analyzed by FACS. CD4$^+$ T cells (red); CD8$^+$ T cells (green); CD19$^+$ B cells (blue); and isotype controls (gray).

A. PD-L2 expression on CD4$^+$, CD8$^+$ and CD19$^+$ TIL

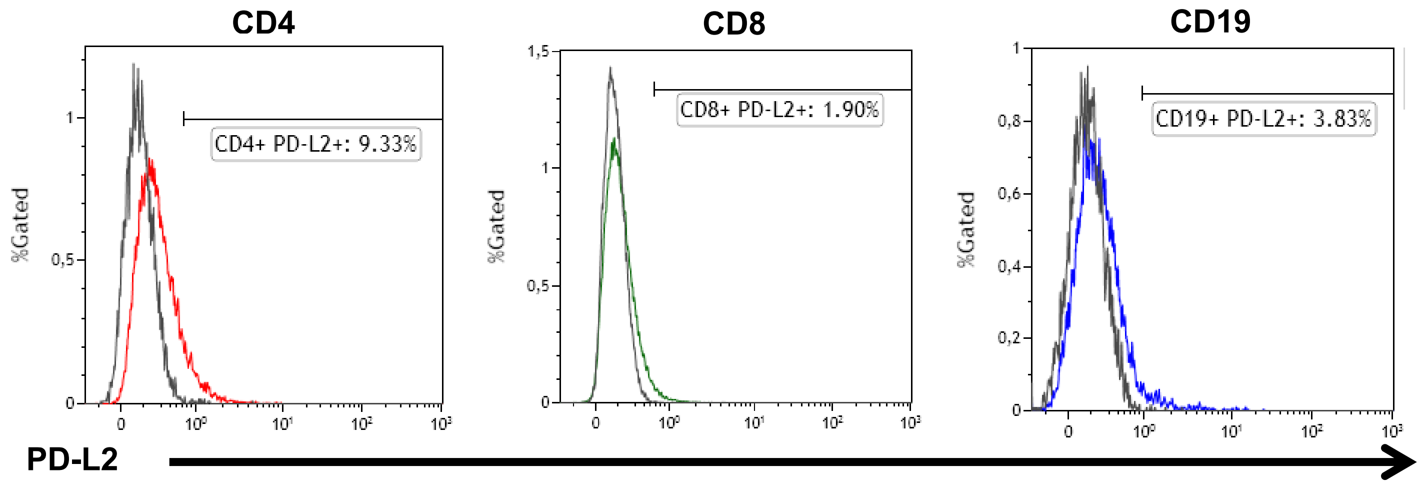

B. PD-L2 expression in human spleen and normal breast (IHC)

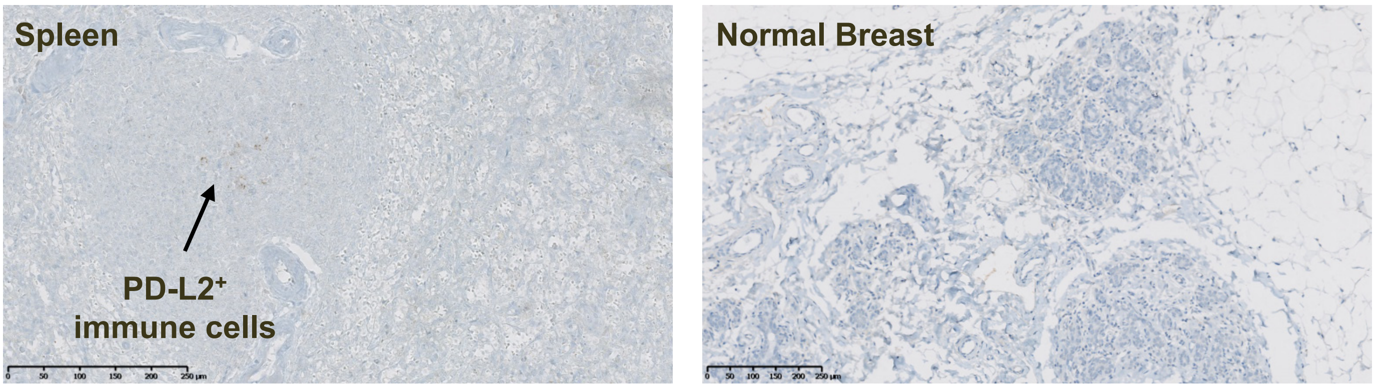

Figure S3: PD-L2 expression on tumor infiltrating lymphocytes (TIL) in breast tumors analyzed by FACS. CD4$^+$ T cells (red); CD8$^+$ T cells (green); CD19$^+$ B cells (blue); and isotype controls (gray). (B) PD-L2 expression (brown) in human spleen (left) (IHC; magnification: 15X) and normal breast tissues (right) (IHC; magnification: 10X).

iCTLA-4 expression on CD4$^+$, CD8$^+$ and CD19$^+$ TIL

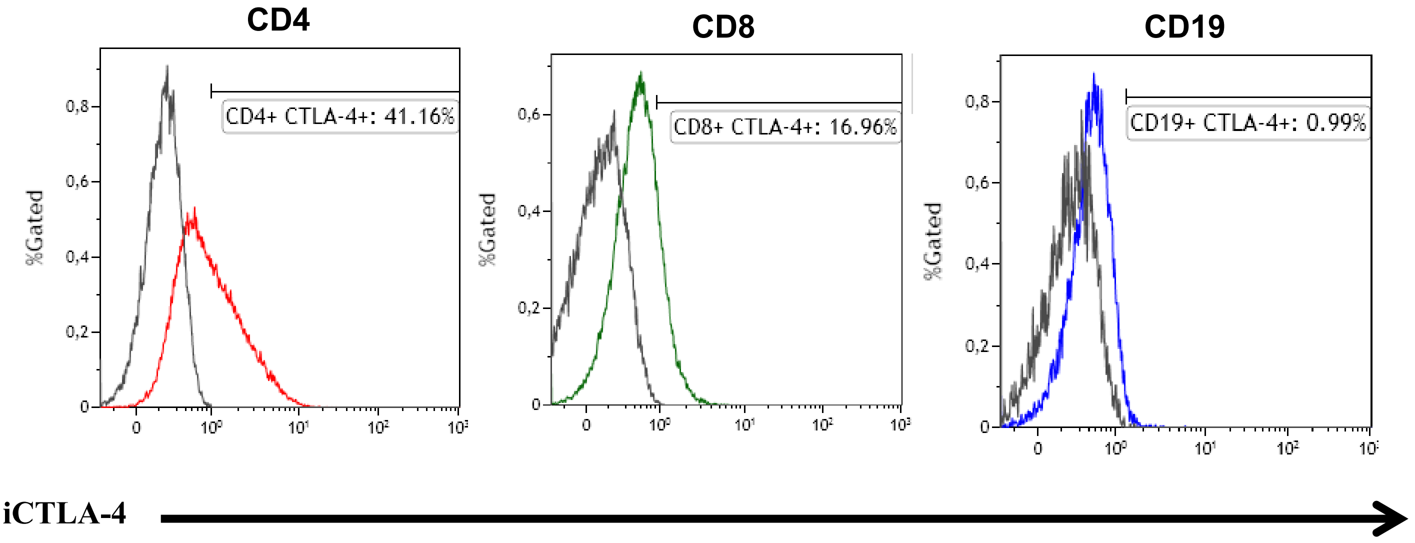

Figure S4: Intracellular CTLA-4 (iCTLA-4) expression on tumor infiltrating lymphocytes (TIL) in breast tumors analyzed by FACS. CD4$^+$ T cells (red); CD8$^+$ T cells (green); CD19$^+$ B cells (blue); and isotype controls (gray).

A. LAG3 expression on $CD4^+$, $CD8^+$ and $CD19^+$ TIL

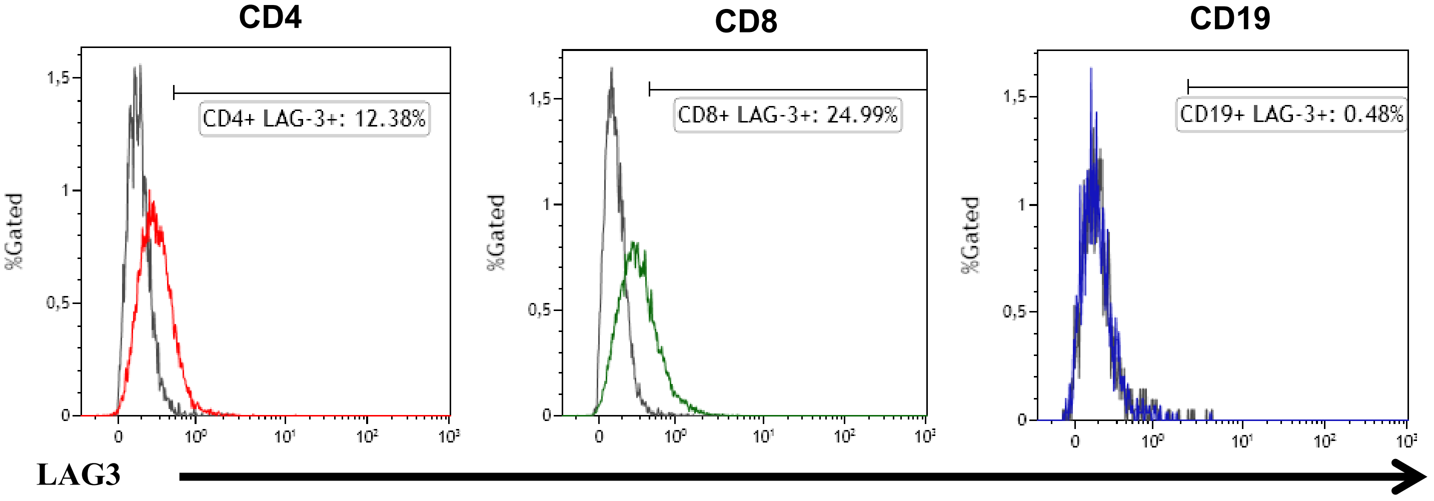

B. LAG3 expression in human tonsil and lymph node tissues

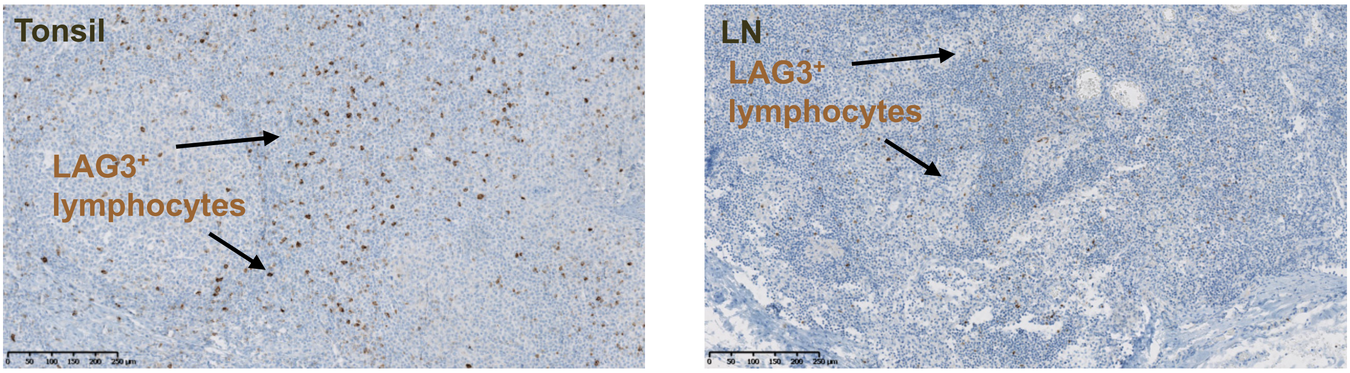

C. LAG3 expression in human tonsil tissue

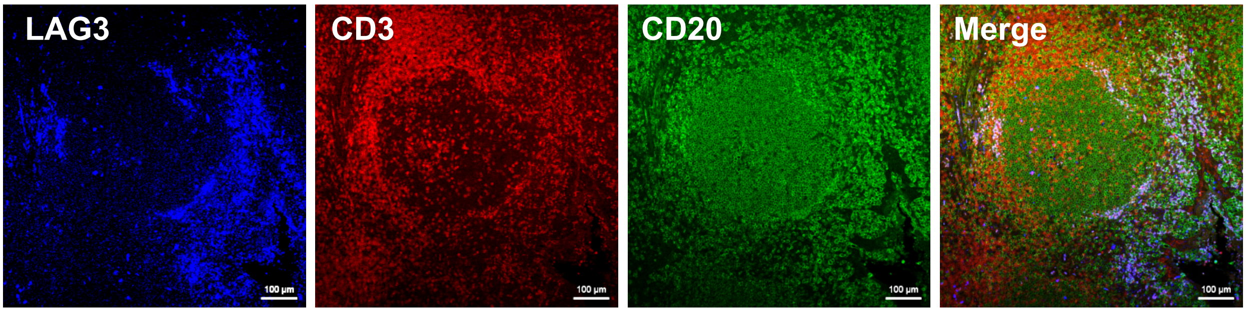

Figure S5: LAG3 expression on tumor infiltrating lymphocytes (TIL) in breast tumors analyzed by FACS. $CD4^+$ T cells (red); $CD8^+$ T cells (green); $CD19^+$ B cells (blue); and isotype controls (gray). (B) IHC staining for LAG3 (brown) in human tonsils and lymph node (LN) tissues. (C) Immunofluorescent staining for LAG3 (blue) together with T (CD3 in red) and B (CD20 in green) cell markers in human tonsil tissue. The merges shows $LAG3^+$ $CD3^+$ cells (pink).

A. TIM3 expression by $CD4^+$, $CD8^+$ and $CD19^+$ TIL

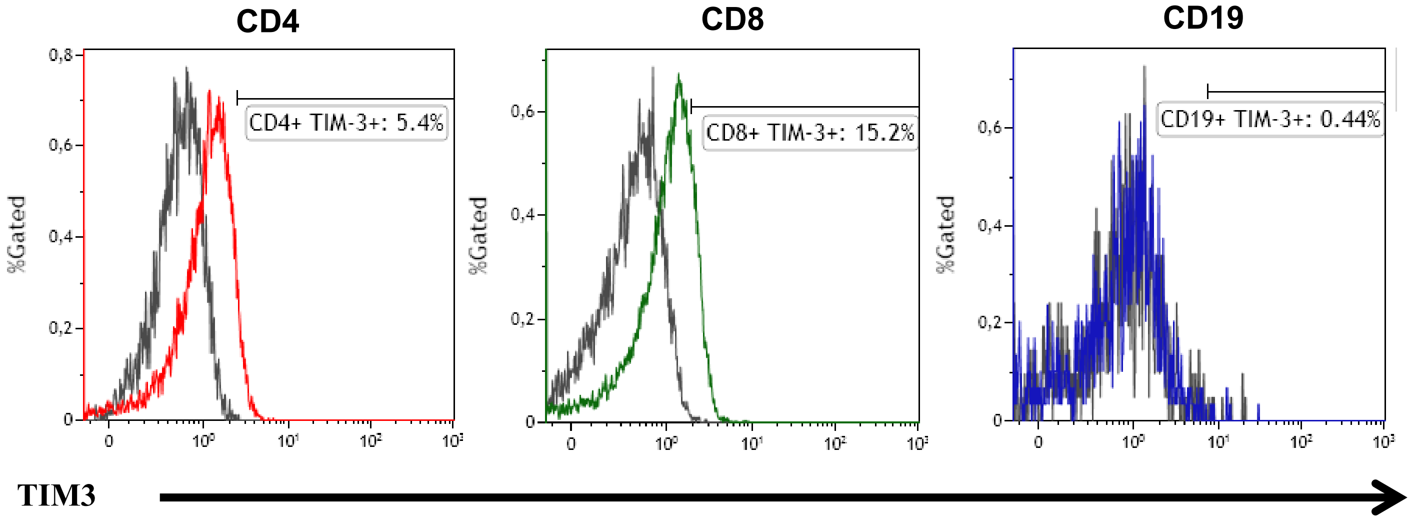

B. TIM3 expression in human tonsil and lymph node

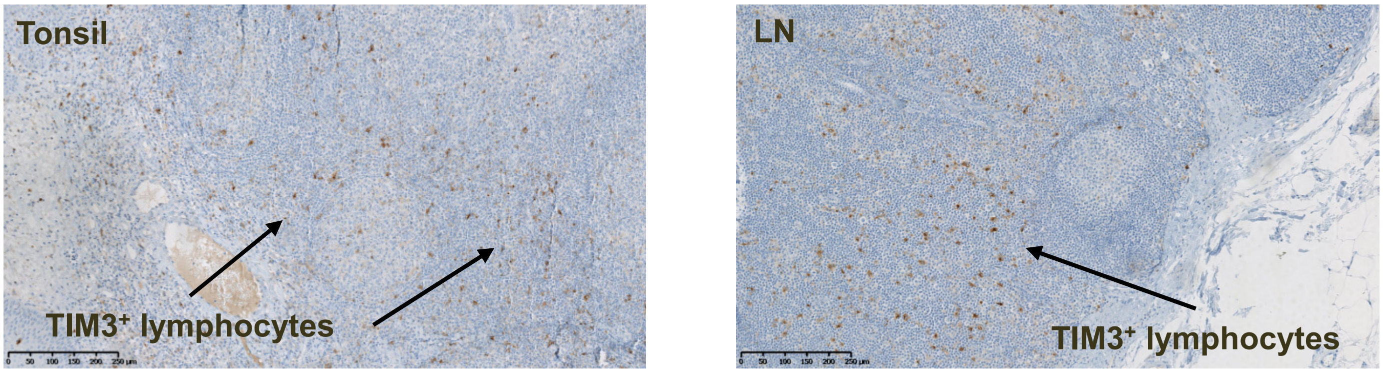

C. TIM3 and CD68 expression in human breast cancer

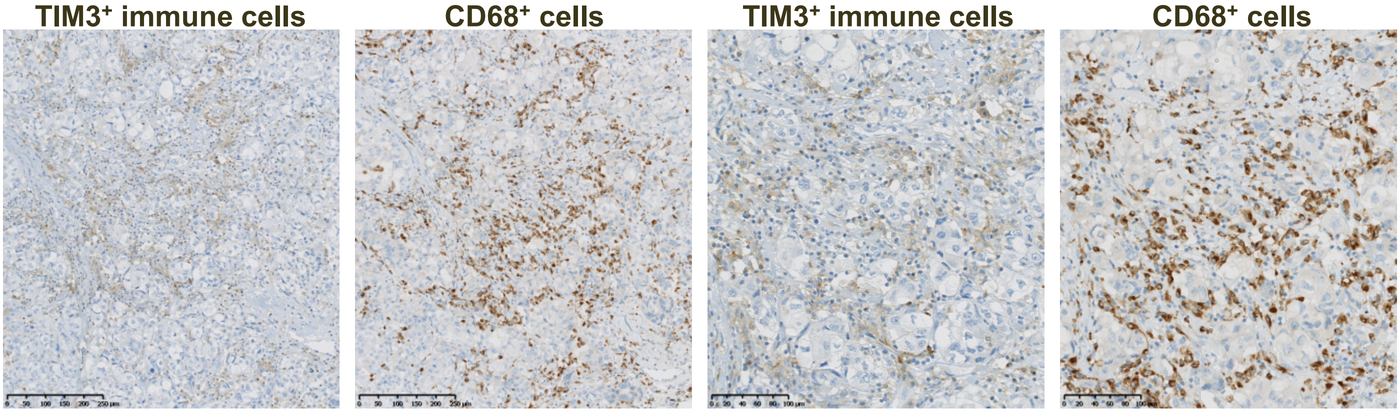

Figure S6: TIM3 expression on tumor infiltrating lymphocytes (TIL) in breast tumors analyzed by FACS. $CD4^+$ T cells (red); $CD8^+$ T cells (green); $CD19^+$ B cells (blue); and isotype controls (gray). (B) IHC staining for TIM3 (brown) in human tonsils and lymph node (LN) tissues. (C) Individual IHC staining of consecutive tissues sections from human breast cancer TIM3 (brown) and CD68 (brown). Magnification 10X (left 2 panels) and 20X (right two panels).

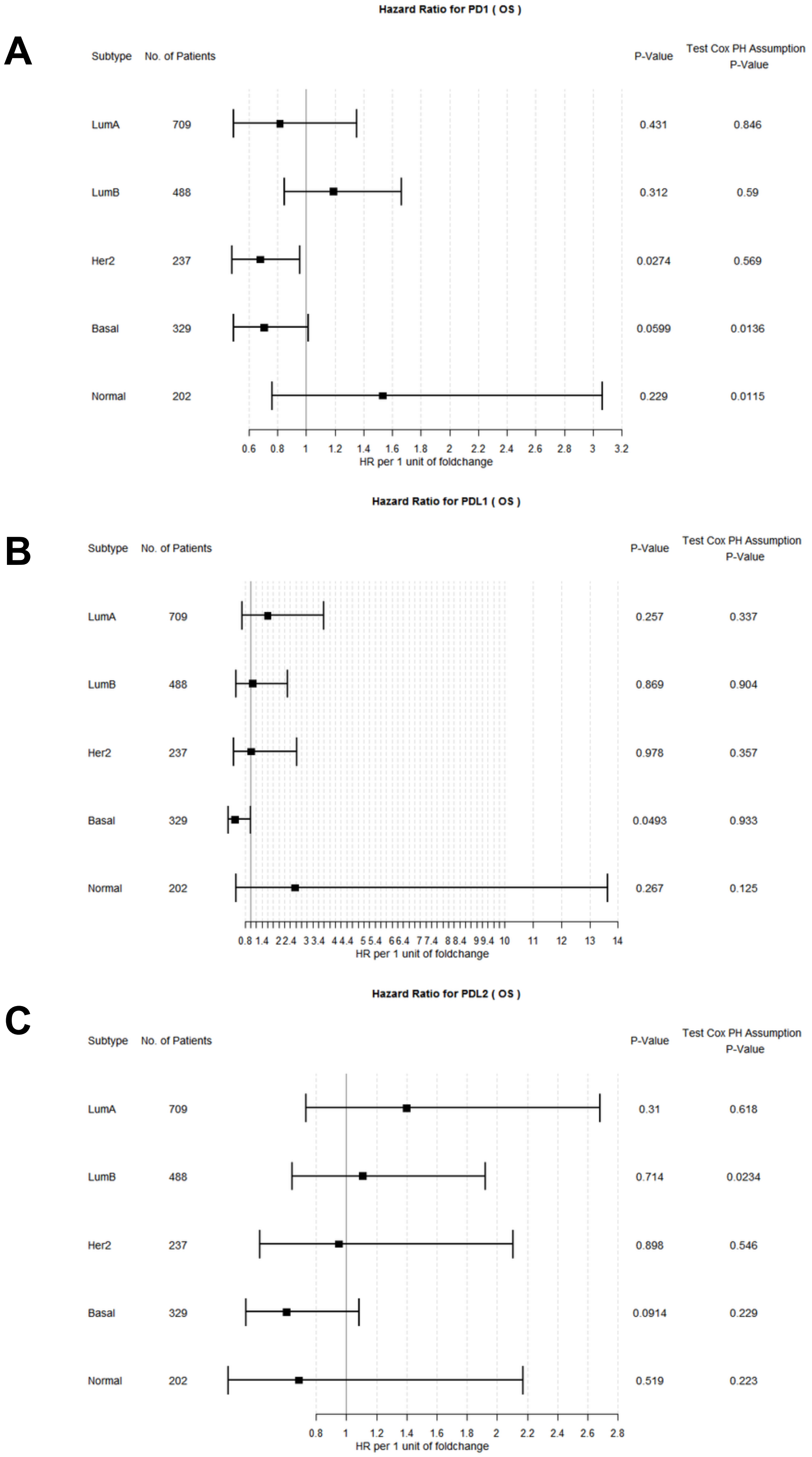

Figure S7: Overall survival (OS) based on gene expression in the METABRIC dataset for: (A) PD-1, (B) PD-L1 and (C) PD-L2 grouped in the PAM50 breast cancer molecular subtypes. Univariate model.

D

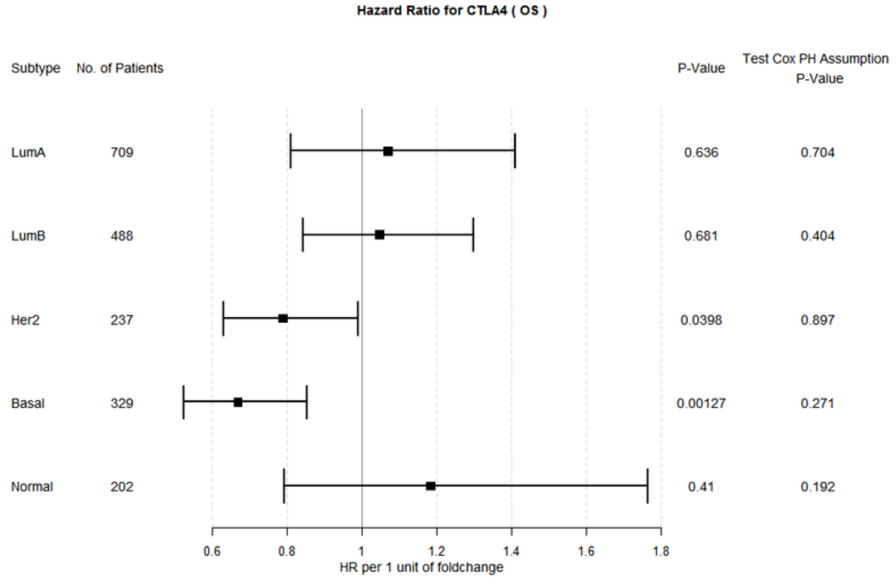

E

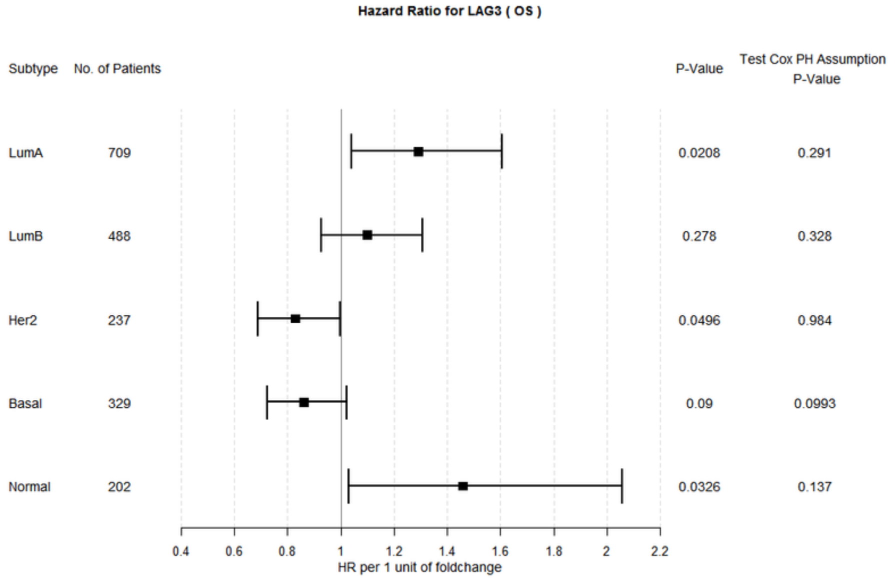

F

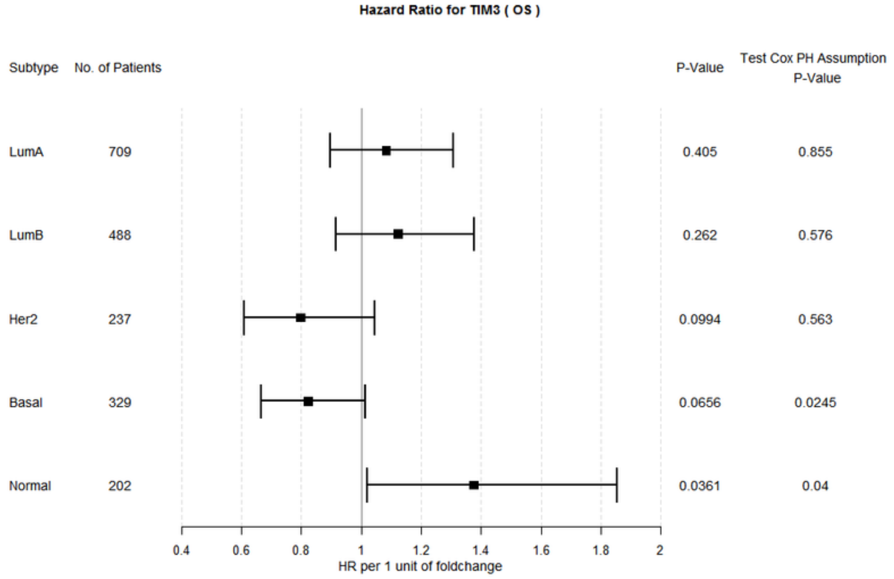

Figure S7 (continued): Overall survival (OS) based on gene expression in the METABRIC dataset for: (D) CTLA-4, (E) LAG3 and (F) TIM3 grouped in the PAM50 breast cancer molecular subtypes. Univariate model.

Figure S8

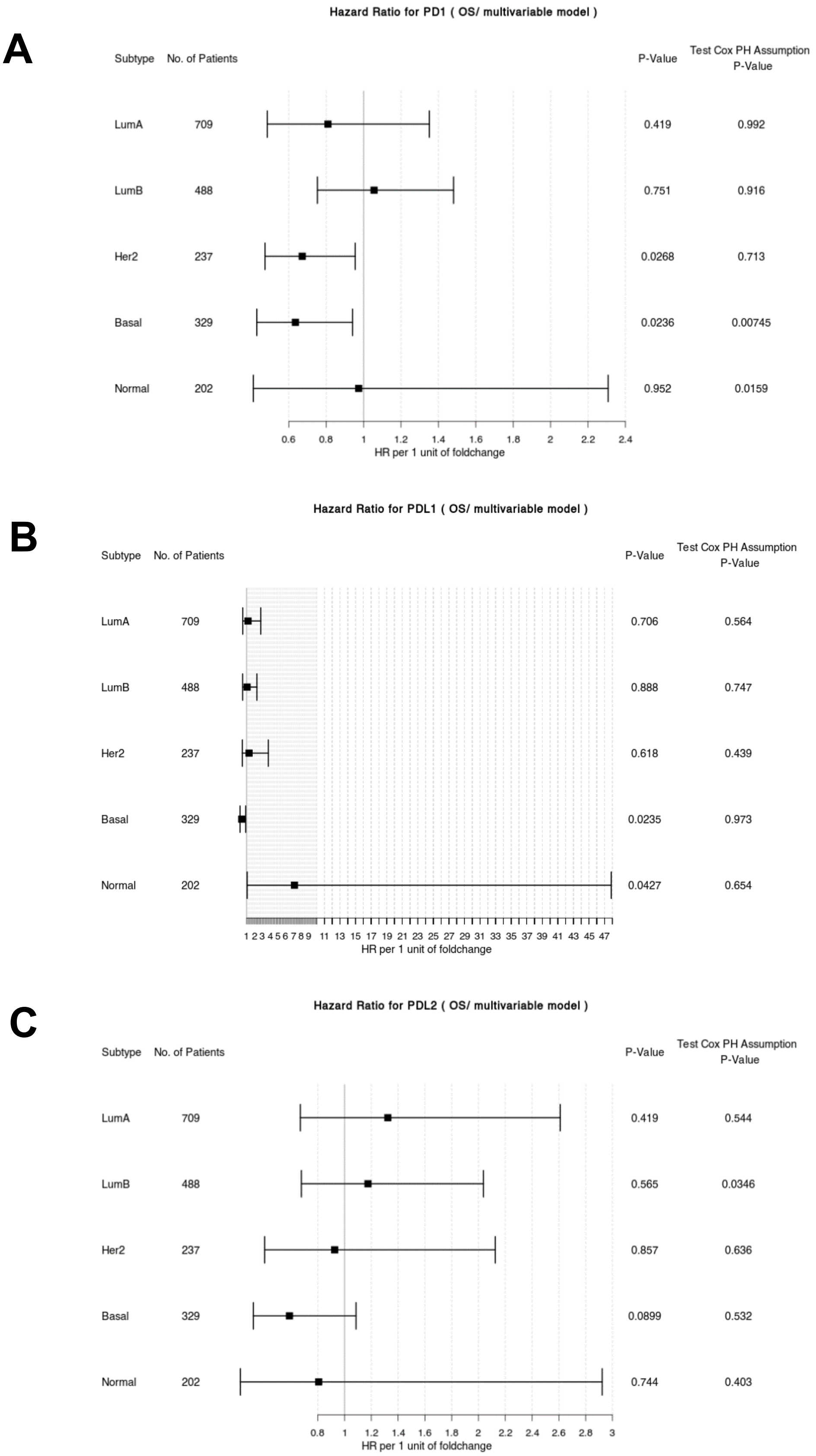

<u>Figure S8</u>: Overall survival (OS) based on gene expression in the METABRIC dataset for: (A) PD-1, (B) PD-L1 and (C) PD-L2 grouped by breast cancer PAM50 molecular subtypes. Multivariable model including: treatment (yes *vs* no), age (≤ 50 *vs* > 50), tumor size (T0, 1, 2 *vs* T3, 4), nodal status (negative *vs* positive), histologic grade (1, 2 *vs* 3), ER status (negative *vs* positive), and human epidermal growth factor receptor 2 (HER2) status (negative *vs* positive).

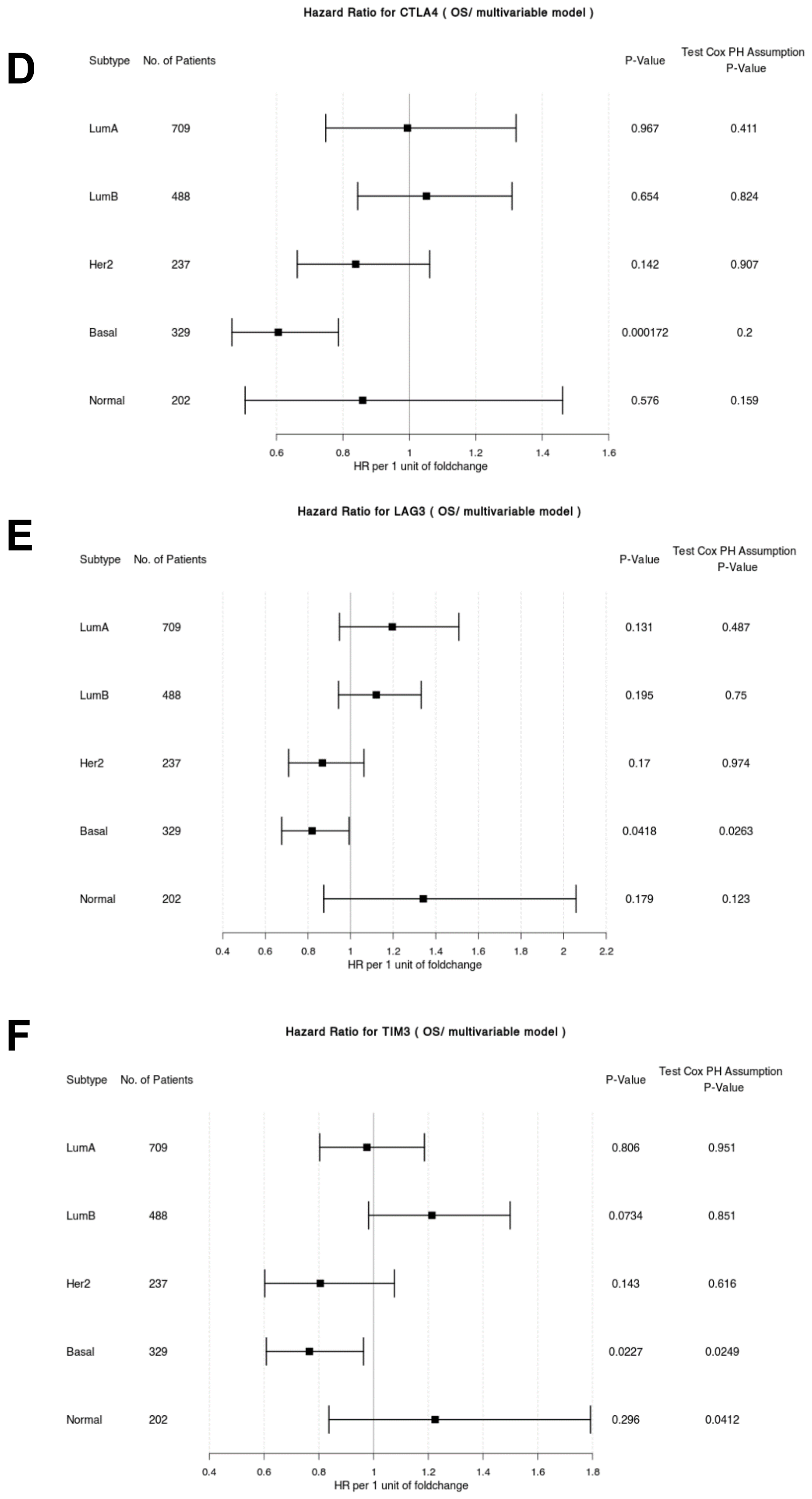

Figure S8 (continued): Overall survival (OS) based on gene expression in the METABRIC dataset for: (D) CTLA-4, (E) LAG3 and (F) TIM3 grouped by breast cancer PAM50 molecular subtypes. Multivariable model including: treatment (yes *vs* no), age (≤ 50 *vs* > 50), tumor size (T0, 1, 2 *vs* T3, 4), nodal status (negative *vs* positive), histologic grade (1, 2 *vs* 3), ER status (negative *vs* positive), and human epidermal growth factor receptor 2 (HER2) status (negative *vs* positive).

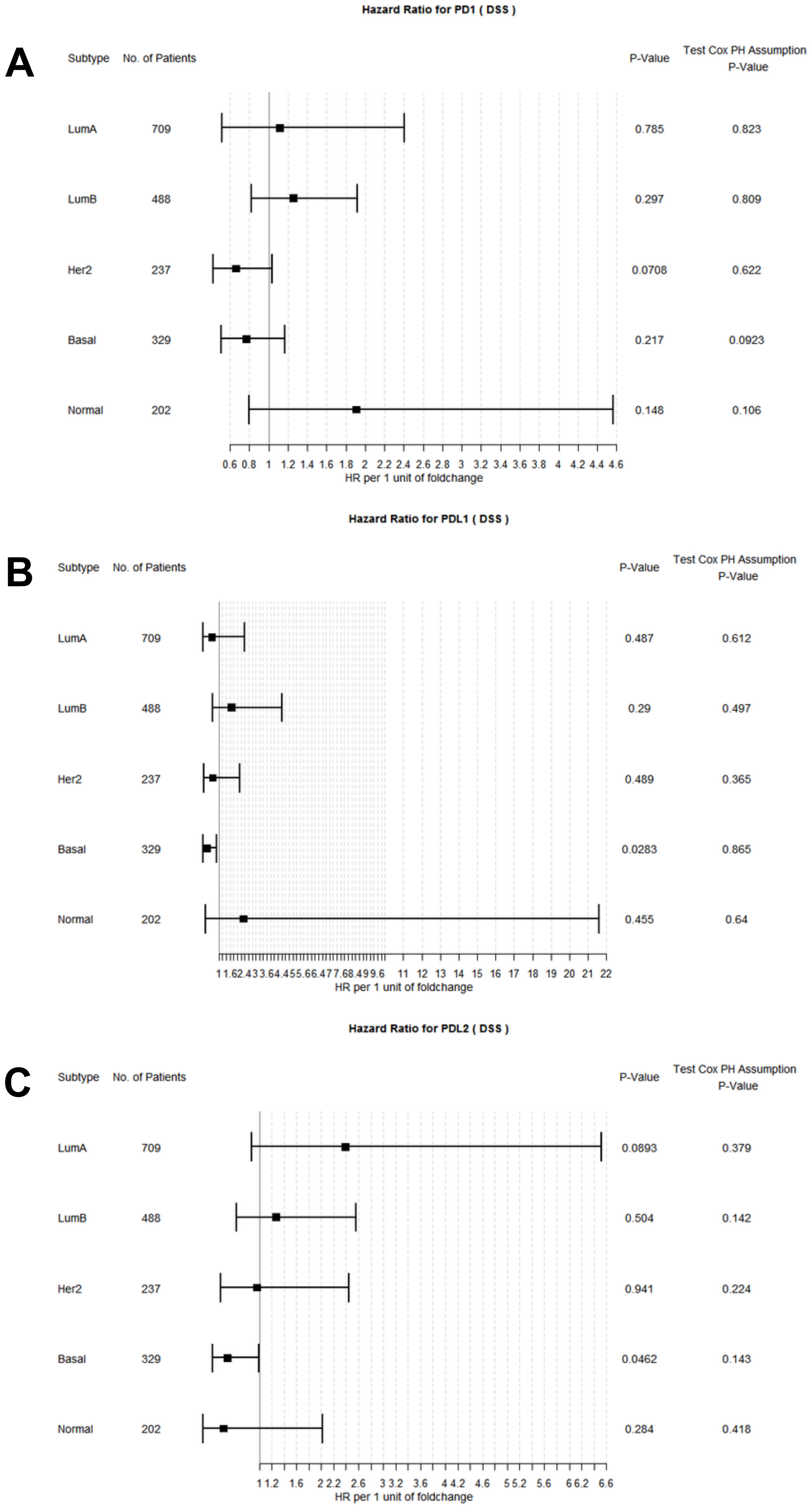

<u>Figure S9</u>: Disease specific survival (DSS) based on gene expression in the METABRIC dataset for: (A) PD-1, (B) PD-L1 and (C) PD-L2 grouped in the breast cancer PAM50 molecular subtypes. Univariate model.

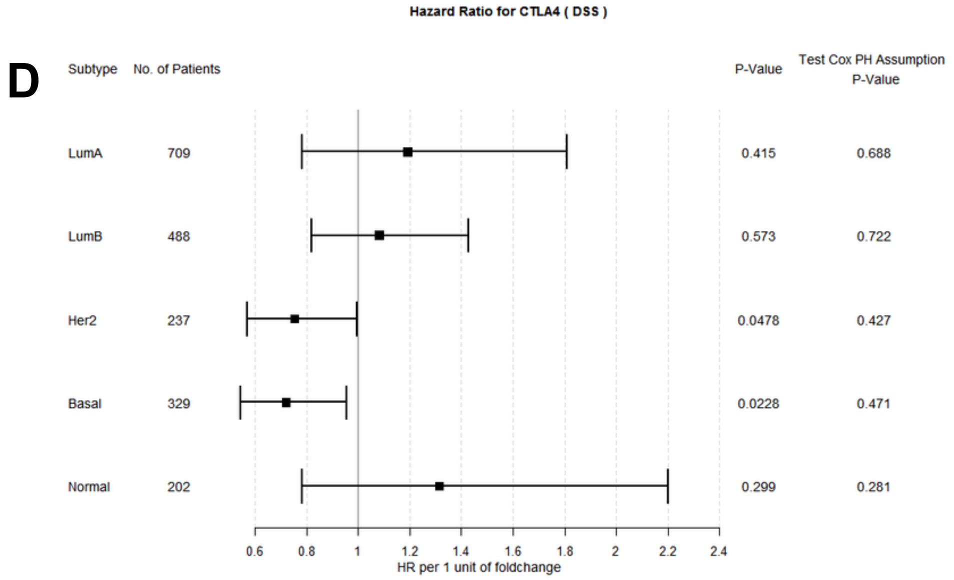

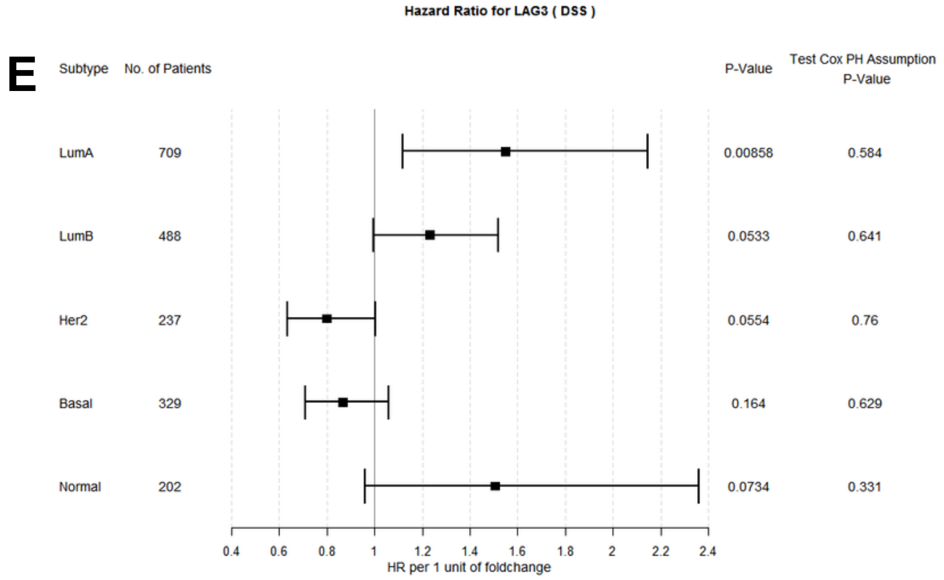

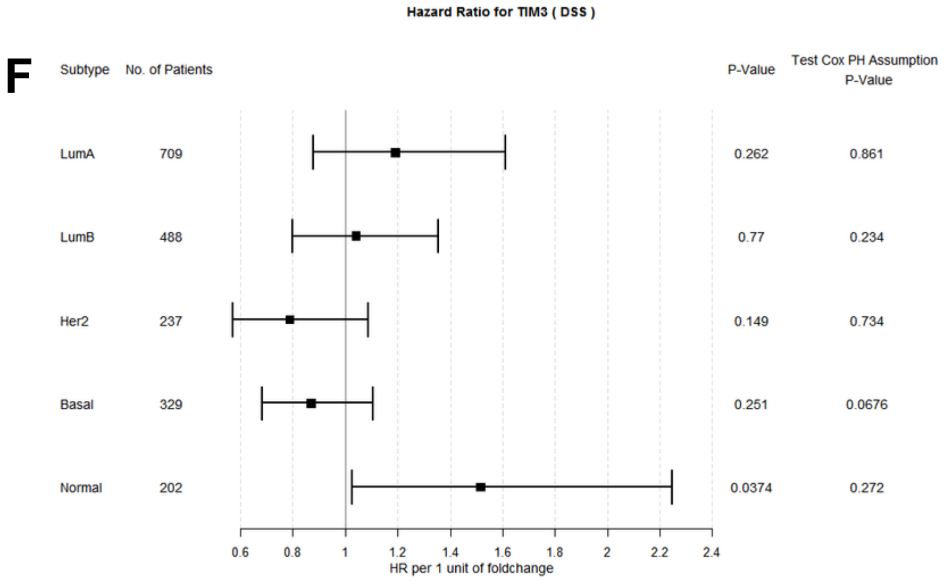

Figure S9 (continued): Disease specific survival (DSS) based on gene expression in the METABRIC dataset for: (D) CTLA-4, (E) LAG3 and (F) TIM3 grouped in the PAM50 breast cancer molecular subtypes. Univariate model.

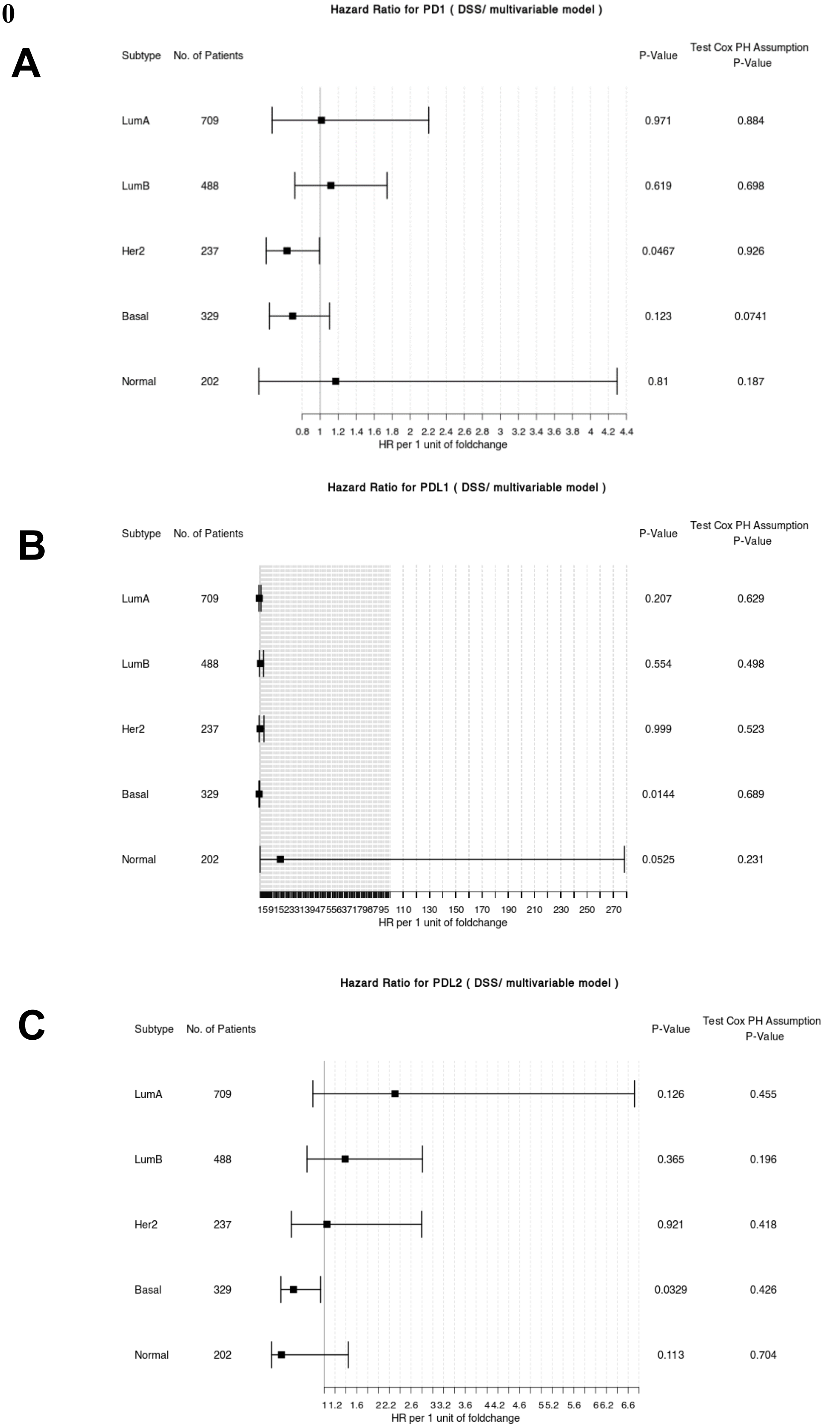

<u>Figure S10</u>: Disease specific survival (DSS) based on gene expression in the METABRIC dataset for: (A) PD-1, (B) PD-L1 and (C) PD-L2 grouped by breast cancer PAM50 molecular subtypes. Multivariable model including: treatment (yes *vs* no), age (≤ 50 *vs* > 50), tumor size (T0, 1, 2 *vs* T3, 4), nodal status (negative *vs* positive), histologic grade (1, 2 *vs* 3), ER status (negative *vs* positive), and human epidermal growth factor receptor 2 (HER2) status (negative *vs* positive).

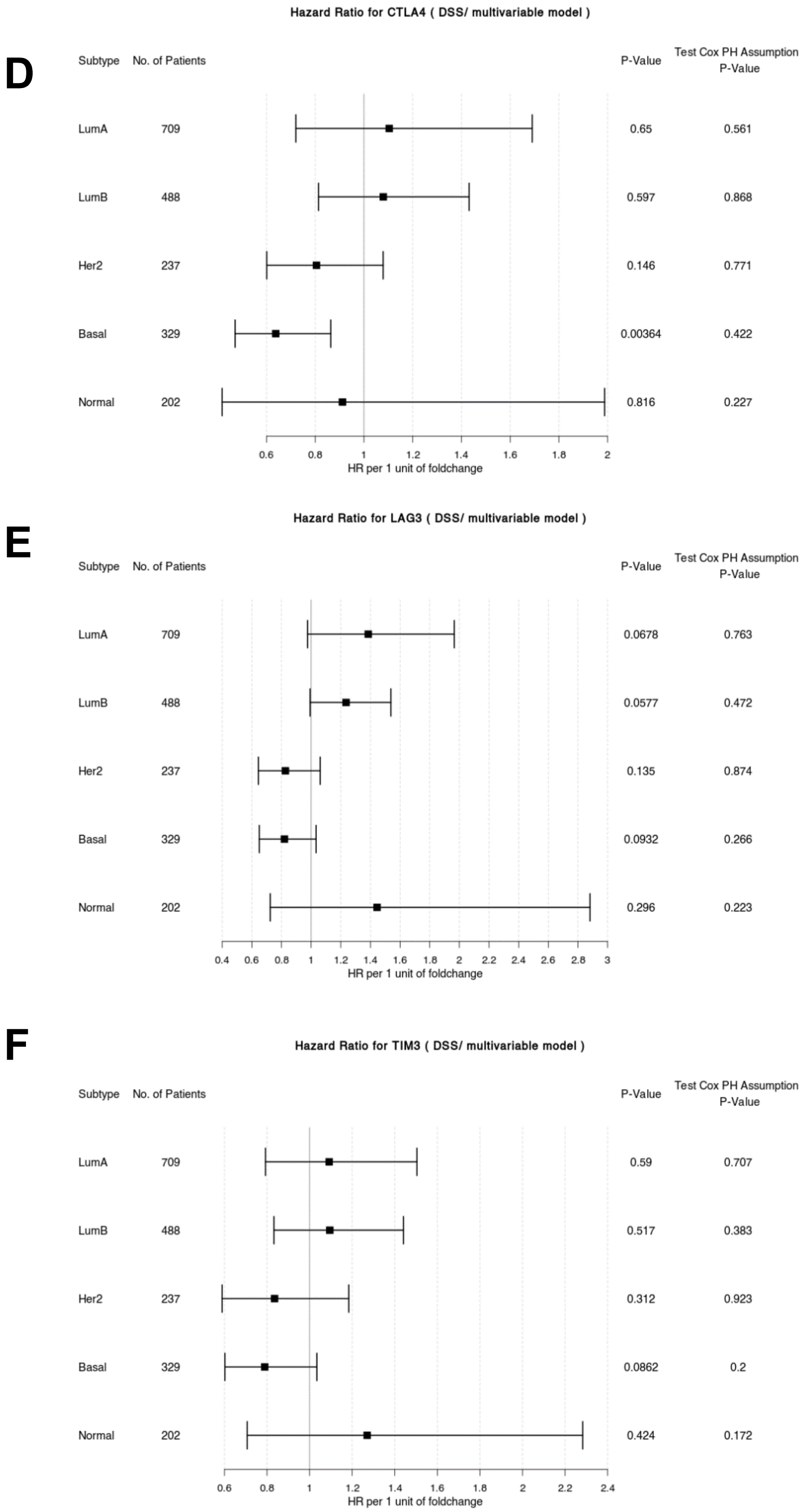

<u>Figure S10 (continued)</u>: Disease specific survival (DSS) based on gene expression in the METABRIC dataset for: (D) CTLA-4, (E) LAG3 and (F) TIM3 grouped by breast cancer PAM50 molecular subtypes. Multivariable model including: treatment (yes *vs* no), age (≤ 50 *vs* > 50), tumor size (T0, 1, 2 *vs* T3, 4), nodal status (negative *vs* positive), histologic grade (1, 2 *vs* 3), ER status (negative *vs* positive), and human epidermal growth factor receptor 2 (HER2) status (negative *vs* positive).
